# Discovery of Novel Sequences in 1,000 Swedish Genomes

**DOI:** 10.1093/molbev/msz176

**Published:** 2019-09-24

**Authors:** Jesper Eisfeldt, Gustaf Mårtensson, Adam Ameur, Daniel Nilsson, Anna Lindstrand

**Affiliations:** 1 Department of Molecular Medicine and Surgery, Center for Molecular Medicine, Karolinska Institute, Stockholm, Sweden; 2 Science for Life Laboratory, Karolinska Institutet Science Park, Solna, Sweden; 3 Department of Clinical Genetics, Karolinska University Hospital, Stockholm, Sweden; 4 Division of Nanobiotechnology, Department of Protein Science, Science for Life Laboratory, School of Engineering Sciences in Chemistry, Biotechnology and Health, KTH Royal Institute of Technology, Stockholm, Sweden; 5 Science for Life Laboratory, Department of Immunology, Genetics and Pathology, Uppsala University, Uppsala, Sweden

**Keywords:** population genomics, novel sequences, de novo assembly, ancestral deletion

## Abstract

Novel sequences (NSs), not present in the human reference genome, are abundant and remain largely unexplored. Here, we utilize de novo assembly to study NS in 1,000 Swedish individuals first sequenced as part of the SweGen project revealing a total of 46 Mb in 61,044 distinct contigs of sequences not present in GRCh38. The contigs were aligned to recently published catalogs of Icelandic and Pan-African NSs, as well as the chimpanzee genome, revealing a great diversity of shared sequences. Analyzing the positioning of NS across the chimpanzee genome, we find that 2,807 NS align confidently within 143 chimpanzee orthologs of human genes. Aligning the whole genome sequencing data to the chimpanzee genome, we discover ancestral NS common throughout the Swedish population. The NSs were searched for repeats and repeat elements: revealing a majority of repetitive sequence (56%), and enrichment of simple repeats (28%) and satellites (15%). Lastly, we align the unmappable reads of a subset of the thousand genomes data to our collection of NS, as well as the previously published Pan-African NS: revealing that both the Swedish and Pan-African NS are widespread, and that the Swedish NSs are largely a subset of the Pan-African NS. Overall, these results highlight the importance of creating a more diverse reference genome and illustrate that significant amounts of the NS may be of ancestral origin.

## Introduction

De novo assembly is arguably one of the most fundamental and perhaps varied tasks of Bioinformatics. Through the use of de novo assembly, short massive parallel sequencing reads are extended into longer continuous sequences (Paszkiewicz and Studholme 2010). These contiguous sequences called contigs are the basis for constructing reference genomes (Lander et al. 2001), transcriptomes (Henschel et al. 2012; Schulz et al. 2012), and for conducting a wide range of analyses, including variant calling (Iqbal et al. 2013; Li 2015). There is a plethora of de novo assemblers (Warren et al. 2007; Butler et al. 2008; Simpson et al. 2009). Most of the de novo assemblers are specialized for a certain task (Li 2015) or data type (Chin et al. 2013). Population-scale de novo assembly projects have provided vast amounts of clinically relevant information, including catalogs of variation and characterization of the major histocompatibility complex haplotypes (Mallick et al. 2016; Maretty et al. 2017).

Compared with mapping assembly, de novo assembly comes with a great advantage: The data may be analyzed independently of the reference genome, which allows for a more detailed analysis of unmappable sequence and regions of low mappability (Chaisson et al. 2015). Unmappable sequences, sometimes known as novel sequences (NSs), are known to be an important contributor to the genetic diversity of human individuals and have been shown to affect phenotypic traits (Kehr et al. 2017).

Similar to other types of variation, NS insertions may act as risk factors in complex disease (Kehr et al. 2017), and they have also been found to be the causative variant in a range of diseases, including dementia (Owen et al. 1992) and cancer (Goossens and Hoshida 2015).

In addition, the study of NS is of importance for understanding the evolution and architecture of the human genome. These sequences may be novel in the sense that they have arisen from events, such as viral insertions or through DNA mutation. However, the vast majority of unmappable sequences are thought to originate from ancestral sequences, missing from the current reference genomes, or deleted within large parts of the population (Mallick et al. 2016; Ameur et al. 2018). Although there is a great interest in NS, these variants are relatively unstudied compared with other types of variation, such as single nucleotide variants (Lek et al. 2016) or copy number variants (Cooper et al. 2011). The study of NS is complicated due to the short read length of the second-generation massive parallel sequencers, as well as the great computational cost of performing de novo assembly on large whole genome sequencing (WGS) data sets.

In this study, we explore the unmappable sequences of 1,000 present day Swedish individuals, representing a cross-section of the population (Ameur et al. 2017). We detect 101 Mb of sequence not present in GRCh37, 46 Mb of which is not present in GRCh38, and therefore to be considered NS. We explore these sequences and show that there is a great diversity among the NS, and that a significant amount of these sequences are found in the Pan-African (Sherman et al. 2019) and Icelandic (Kehr et al. 2017) populations, as well as in chimpanzee reference genome. Although the NSs are largely repetitive, they also consist of significant amounts of sequence not masked by the RepeatMasker tool (http://www.repeatmasker.org/; last accessed August 12, 2019).

## Results

### De Novo Assembly of the SweGen Cohort

**Fig. 1.**
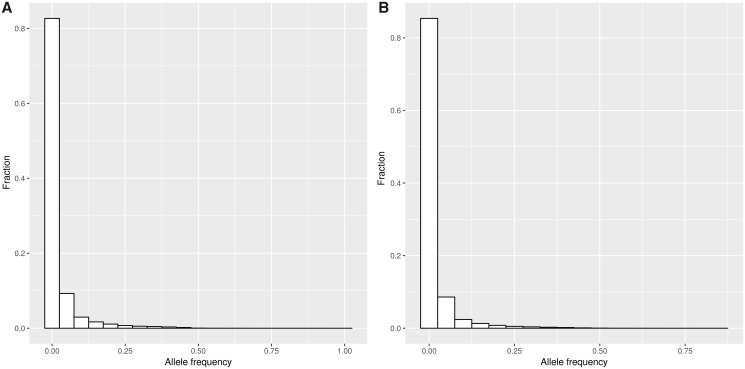
Frequencies of GRCh37UC and NS. Histograms displaying the fraction of GRCh37UC (*A*) and NS (*B*) present at various frequencies across the population.

All 1,000 genomes of the SweGen cohort were successfully assembled using the Assemblatron workflow (table 1). The resulting assemblies are relatively large in size (4,109 Mb in average) compared with the GRCh37 reference genome (about 3,000 Mb). The large assembly size is likely due to the formation and retention of “bubbles” at heterozygote variant sites, which increases the assembly size, but decreases the contiguousness (table 1). On average, the genome wide coverage of the assemblies is 88.85%, which is high considering that 7.6% of GRCh37 consists of N and that half the population is female, and will therefore lack coverage across chromosome Y. On average, each individual genome harbored 8 Mb of unmappable genetic sequence prior to filtering (table 1 and supplementary table S1, Supplementary Material online), distributed among 36,159 contigs per individual on average. After removal of contaminants and small contigs (<301 bp), each individual carry, on average, 1.8 Mb of contigs unmappable to GRCh37 (GRCh37UC), distributed among, on average, 3,600 contigs. Thirty-eight percent of these GRCh37UC do not match significantly to GRCh38 (identity >80%, coverage >50%) and is therefore to be considered NS; resulting, on average, 1,362 NSs per individual, distributed among, on average, 0.6 Mb. The GRCh37UC were clustered using CD-hit, producing 126,885 contig clusters, totaling 101 Mb of sequence. Eighty-nine percent (112,955 clusters) of the GRCh37UCs are rare (allele frequency < 5%), and no contig cluster was found in all individuals (fig. 1*A*) . The NSs are a subset of the GRCh37UC, consisting of 61,044 clusters (totaling 46 Mb) that do not have any significant match across the GRCh38 reference genome. Performing the same analyses with only the NS, we found that 91% (55,395 clusters) of the NSs are rare (fig. 1*B*).

**Table 1.** Performance of Assemblatron in the SweGen Data Set.

	Average	Standard Deviation
Assembly size (Mb)	4,109	1,095
N50 (bp)	1,006	81
L50	665,344	46,071
N90 (bp)	251	3
L90	5,319,171	304,717
Unfiltered and unaligned sequence (kb)	7,800	1,786
GRCh37UC (kb)	1,758	165
Novel sequence (kb)	614	102
Coverage across GRCh37 (%)	88.85	0.62

Note.—Mb, mega base pairs; bp, base pairs; kb, kilo base pairs.

**Fig. 2.**
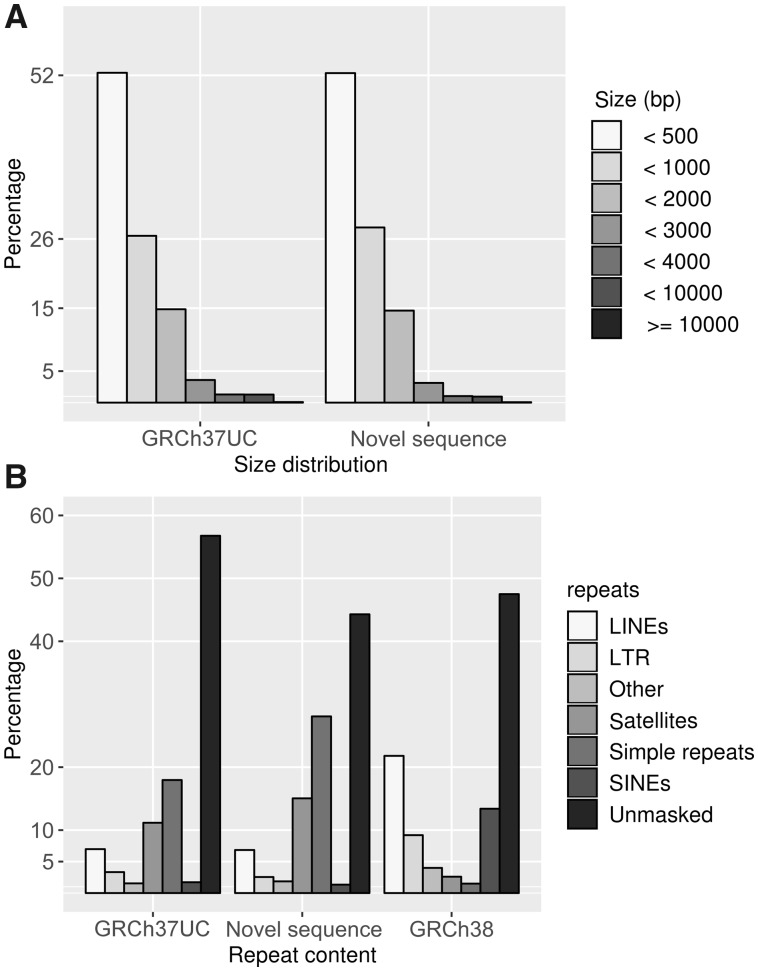
Characteristics of the GRCh37UC and NS. (*A*) A size histogram of the quality controlled GRCh37UC and NS. (*B*) A summary of the RepeatMasker analysis of the GRCh37UC, NS, and randomly selected regions of GRCh38; each bar display the fraction of a specific RepeatMasker repeat class, the “other” bar includes multiple classes, including RNA and low complexity sequence.

Comparing the size distributions of the filtered GRCh37UC and NS, we find that they are similar in size, and that these sequences span the same size range (301–22,701 bp) (fig. 2*A*). Notably, the size ranges were considerably wider prior to filtering: The smallest unmappable contig was sized 73 bp, and all contigs <301 bp were removed by the size filter, and the largest contigs sized (22.7–60 kb) were all classified as contaminant sequence, mainly of viral origin.

The 126,885 GRCh37UC and 61,044 NS clusters were analyzed for repeat elements using RepeatMasker. Notably, the GRCh37UC contain a higher percentage of unmasked sequence compared with the GRCh38 reference genome (fig. 2*B*) Additionally, the GRCh37UC consists of a larger percentage of simple repeats and satellites compared with GRCh38. On the other hand, the GRCh37UC contains significantly lower amounts of long interspersed nuclear elements (LINEs), long terminal repeats, and short interspersed nuclear elements (SINEs), the estimated *P* value of all repeat classes was *P* < 10^−4^.

Likewise, the repeat content of the NS differs from GRCh38: The NS consists of a smaller percentage of unmasked sequence but is enriched in satellite and simple repeats, and the estimated *P* value of all repeat classes was *P* < 10^−4^. With the exception of the unmasked sequences, the repeat content distribution of NS and GRCh37UC follow similar patterns: Compared with GRCh38, they are both enriched in simple repeats and satellites, but they are both relatively lacking in other classes of repeat elements (fig. 2*B* and supplementary data set S1, Supplementary Material online).

**Fig. 3.**
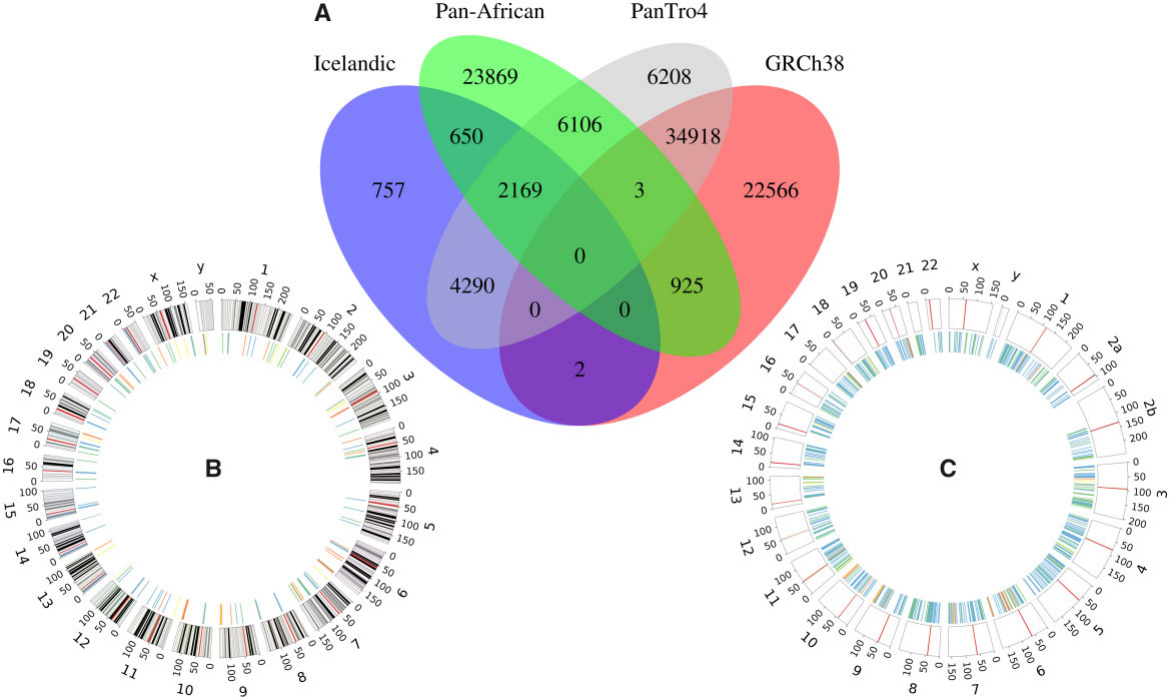
Alignment of the GRCh37UC. (*A*) The number of GRCh37UC clusters mapping confidently to GRCh38, Pan_tro 4.0 (PT4), the Pan-African NS, and an Icelandic NS catalog. (*B*, *C*) The distributions of GRCh37UC across GRCh38 and PT4. The colors of the inner circle indicate the percentage density of contig clusters within 3-Mb sized bins (white = 0%, blue < 0.1%, green < 0.5%, yellow < 1%, orange <5%, red < 10%, purple < 20%, and 20% => black).

Next, the GRCh37UC were aligned to GRCh38, PanTro 4.0 (PT4), Pan-African NS (Sherman et al. 2019), and a catalog of Icelandic NS (Kehr et al. 2017). Eighty percent of the GRCh37UC aligned confidently (identity >95%; coverage >90%) to any of these references/catalogs with 58414 GRCh37UC clusters aligning to GRCh38 (fig. 3*A*); 53,692 GRCh37UC clusters aligned to PT4, however the majority of these sequences (34,921) also align to GRCh38. 33722 GRCh37UC clusters align to the Pan-African NS catalog, and only 928 of these sequences align to GRCh38: indicating that there’s a great amount of NS shared between the Pan-African and Swedish population. A total of 7,868 GRCh37UC clusters align to the Icelandic NS, however most of these sequences align to another reference genome/NS catalog as well (fig. 2*A*). NSs (42,684, 70%) aligned confidently to any of these data sets, and 48,357 NSs (79%) fulfilled the alignment threshold of 80% identity and 50% coverage.

In total, 77,187 of the 126,885 GRCh37UC clusters were aligned confidently to either GRCh38 or PT4, allowing for the analysis of the positioning of these sequences, as well as comparisons between the two reference genomes. It was found that the GRCh37UC are distributed differently along GRCh38 (fig. 3*B*) and PT4 (fig. 3*C*). In GRCh38, the majority of GRCh37UC align to centromeric and repetitive regions, and the majority (89%) of the 3-Mb bins are devoid of any GRCh37UC. In contrast, there is a more uniform coverage of GRCh37UC across PT4 with 45% of the bins containing a GRCh37UC cluster, indicating that these individuals carry a great diversity of sequences homologous to the chimpanzee.

### Positioning of the Unmappable Sequences

The positioning of the sequences that could not be confidently aligned to GRCh38 was analyzed using two methods: the RetroSeq insertion detection software, and through alignment to PT4.

Briefly, RetroSeq was run using all the GRCh37UC clusters as a sequence library. Insertions of sequences confidently aligning to GRCh38, and insertions present in <5 individuals were removed.

The RetroSeq software detected 4,928 insertions of NS across the GRCh37 reference genome. Notably, these 4,928 insertions involve only 246 NSs; indicating that a number of these sequences represent collapsed repeats rather than a single allele. Out of the 246 NSs, 156 where involved in a single insertion event, and the most recurring NS was inserted at 2,367 genomic positions, this NS is 4,135-bp long, consisting of 45% repeat sequence, including a 994 bp (CA)n repeat; and may therefore represent insertion a variety of (CA)n repeats rather than insertions of that unique NS. The positions of the insertions were searched for overlaps with various genomic features, including centromeres, segmental duplications, genes, and exons, as well as Mendelian inheritance in man (MIM) morbid genes and exons (table 2); briefly, MIM morbid genes are genes reported to be associated with disease in the Online Mendelian Inheritance of Man database (Hamosh et al. 2015). It was found that there’s an enrichment of NS insertions within the repeat regions of the repeat masker bed file. In contrast, there was a significant depletion of insertions within the other features (centromeres, ENSMBL genes, exons, and MIM morbid genes and exons); such depletion could appear due to the conservation of sequences of biological importance (such as the MIM morbid genes), but could also be due to low mappability in some of these regions. In total, we find 2,195 NS insertion events within the ENSMBL genes, out of these 2,195 NS insertions, 337 are positioned within MIM morbid genes. The 2,195 NS insertions are positioned within 2,384 genes, 245 of which are MIM morbid genes; notably, a few genes overlap, and therefore produce a larger number of affected genes compared with the number of insertion events. Among the affected MIM morbid genes, a few are well known to be highly variable, including *HLA-DRB1* that contain a single exonic insertion found in 86 individuals, other genes such as *SOX18* are perhaps less known to undergo rearrangements (supplementary table S2, Supplementary Material online). Eighty-five NS insertion events were found within the exons of 82 genes. Ten of these 85 insertions occurred within 10 MIM morbid genes. Two of these NS insertions were uniquely positioned. One of the uniquely positioned insertions was found within exon 3 of *HLA-DRB1*, and the other uniquely positioned NS was found within *WDR72* exon 20. The *HLA-DRB1* insertion is found in 86 individuals, and the *WDR72* insertion is found in 56 individuals, both of these insertions are therefore to be considered common.

**Table 2.** A Summary of the RetroSeq Analysis.

Feature	Unique NS	Observed Insertion Events	Simulated Insertion Events (%)	*P* Value
Centromere	27	274 (5.6%)	12	<2.2 × 10^−16^
Exons	17	85 (1.7%)	4	<2.2 × 10^−16^
MIM morbid exons	4	10 (0.2%)	0.6	<2.2 × 10^−16^
Segmental duplication	51	283 (5.7%)	5	0.003
ENSMBL genes	122	2,195 (45.6%)	48	1 × 10^−6^
MIM morbid genes	25	337 (6.8%)	8	0.002
Repeat elements	177	3,025 (61.4%)	46	<2.2 × 10^−16^
GRCh37	246	4,928 (100%)	100	—

Note.—Each feature represents the features of various bed files downloaded from ENSMBL Biomart or UCSC Tablebrowser. Unique NSs represent the number of unique NSs inserted in these features, whereas observed insertion events present the total number of insertion events. The simulated insertion events present the percentage of the randomly positioned insertions intersecting the various features. The *P* value column displays the *P* values computed from binomial tests.

Eight NS insertions found within the exons of MIM morbid genes (*SOX18*, *SFTPB*, *C7*, *OPLAH*, *IGF2*, *PACS1*, *KCNC3*, and *AP4S1*) were not uniquely positioned. These insertion calls may therefore represent other events or misalignment rather than true NS insertions (supplementary table S2, Supplementary Material online). The NS insertion event within *AP4S*, was found in 53 individuals (5.3%), and is therefore to be considered common; the remaining events were found in <5% of the individuals.

GRCh37UC clusters (18,773) were confidently aligned to the chimpanzee genome (identity >95%, coverage >90%), but not to GRCh38 (fig. 3*A*). These 18,773 sequence clusters consist of 70% unmasked bases, as well as 17% LINE elements (supplementary data set S1, Supplementary Material online), which stands in stark contrast to the overall NS that are enriched by satellites and simple repeats (fig. 2*B*). Compared with the RetroSeq analysis, 98% of these sequences map uniquely to PT4, and the most recurring sequence align confidently to five genomic positions. Similar to the RetroSeq analysis, it was found that there was a significant enrichment of NS mapping to repeat elements, and depletion of NS aligning within genes and exons (table 3).

**Table 3.** A Summary of the Chimpanzee Genomic Feature Analysis.

Feature	NS	Simulated Alignments (%)	*P* Value
Human gene ortholog	2,807 (15%)	31	<2.2 × 10^−16^
Exons of human gene orthologs	29 (0.16%)	1.7	<2.2 × 10^−16^
Repeat elements	13,074 (69.4%)	47	<2.2 × 10^−16^
PT4	18,773 (100%)	100	—

Note.—Each feature represents the features of various bed files downloaded from ENSMBL Biomart or UCSC Tablebrowser. The NS column represents the number of NSs positioned in each genomic feature. The simulated insertion events present the percentage of the randomly positioned insertions intersecting the various features. The *P* value column displays the *P* values computed from binomial tests. The human gene ortholog features consist of chimpanzee genes having a human gene ortholog, and the exons of human gene orthologs present the NS positioned within the exons of those genes.

**Fig. 4.**
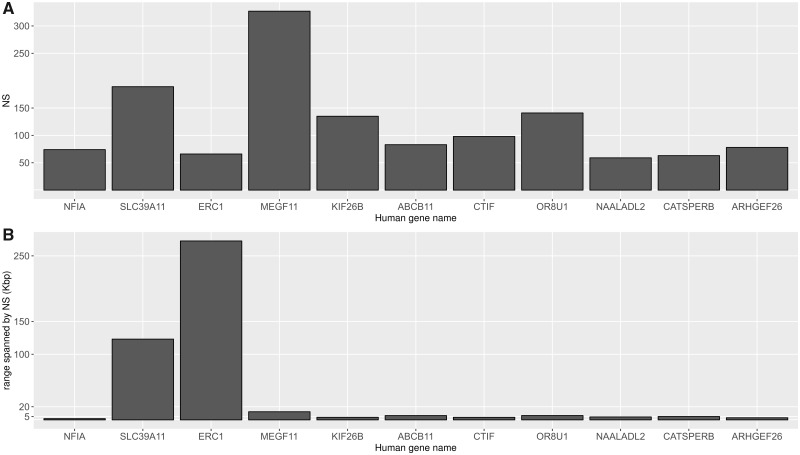
Summary of the top ten most NS enriched chimpanzee genes having a human ortholog. (*A*) A barplot showing the number of NS positioned within the top ten genes. (*B*) The size of the intergenic region (kb) spanned by NS.

NSs (2,807) were confidently positioned within chimpanzee genes having a human gene ortholog (table 3). However, these 2,807 NS are positioned within only 143 genes. Twenty of these genes contain one single NS, and the median number of NS within these 143 genes is 6. The largest number of NS was found in the *MEGF11* gene, harboring 327 unique NS (supplementary table S3, Supplementary Material online), and 1,313 (47%) of these 2,807 NS were found in the 10 most NS enriched genes (fig. 4*A*). Analyzing the spread of the positions of NS within these ten genes, we found two distinct patterns, the NS may be localized within a small region, indicative of hotspots, or they may be widely spread across the gene (fig. 4*B*). For instance, we found that the 74 NS of *NFIA* are contained within a 1.8-kb region, which is small considering that *NFIA* is 385-kb long. In contrast, we found a wider spread of NS across the *ERC1* gene, where 66 NS are spread across a region of 272 kb, which is a significant proportion of the *ERC1* gene (495 kb). In total, we found 29 NS mapping to the exons of chimpanzee genes with a human gene ortholog (table 3) and altogether these 29 NS affect the exons of four genes (*EPPK1*, *OR8U1*, *NINL*, and *METTL21C*). However, the patterns are different exemplified by four NS that map to exon 1 of *NINL*, whereas only one NS overlap with exon 1 of *OR8U1*. Furthermore, we observe a complex pattern of NS aligning to the exons of *EPPK1*, with 19 NS of various length overlapping different exons of chimpanzee *EPPK1*. Compared with human *EPPK1*, which contains 2 exons, the chimpanzee *EPPK1* consist of 53 exons and the NS observed in the SweGen data seems to contain some of these ancestral exons.

A large number of the NS mapping to the chimpanzee genome overlap, forming clusters of similar NS. We merged all uniquely mapped NS using BEDTools merge, resulting in 1,065 distinct clusters, produced from a total of 18,492 uniquely aligned NS. The median number of individuals per cluster is 6 (i.e., 0.06%). Interestingly, the most prevalent NS cluster is found in 353 individuals and 22% (231) of the clusters are personal (only found in a single individual).

Next, the WGS data of 96 individuals was aligned to the PT4 reference genome and we manually inspected 10 random regions containing NS using Interactive genomics viewer (IGV) (Robinson et al. 2011) (supplementary figs. S1–S10, Supplementary Material online). We found that these sequences are present in the chimpanzee genome and hence their absence in GRCh38 represents deletion events relative to the chimpanzee genome. The deletions are consistently longer than the NS contigs (supplementary table S4, Supplementary Material online). Inspecting these deleted regions in IGV, we found that the carriers of these specific NS carry at least one copy of the sequence deleted in the human reference genome (supplementary figs. S1–S10, Supplementary Material online), indicating that the NS are fragmented.

We then analyzed the breakpoint junction (BPJ) characteristics of the same ten GRCh38 deletions relative to the chimpanzee genome (table 4). Matched repeat elements were observed at the BPJs of the deletions overlapping the NS SweGen0010_1572, SweGen0073_3967, and SweGen0005_14992. In contrast, no repeat elements flanked the NS SweGen0068_5778, SweGen0068_3755, SweGen0070_3823, and SweGen0010_5023, instead we found blunt-ends, small insertions or short stretches (one nucleotide) of microhomology at the BPJs. Finally, we observed a five nucleotide stretch of microhomology at the BPJ of the deletion spanning NS SweGen0001_4740 (table 4).

**Table 4.** BPJ Characteristics of Ten Randomly Selected GRCh38 Deletion Events Relative to the Chimpanzee Genome.

NS	Deletion Length (kb)	Position (PT4)	Repeat Start	Repeat End	Breakpoint Microhomology	Breakpoint Insertion
SweGen0068_5778	3	Chr2B: 180318426–180321361	Simple tandem repeat	*ERVL*	C	—
SweGen0068_3755	3	Chr1: 226893660–226896451	*Alu*	—	—	AT
SweGen0010_1572	4 kb	Chr8: 30906438–30910162	*L1*	*L1*	NA	NA
SweGen0073_3967	2 kb	Chr1: 123806530–123808453	*Alu*	*Alu*	GGGTTCAAGT	—
SweGen0005_14992	1 kb	Chr4: 189326049–189327131	*Alu*	*Alu*	NA	NA
SweGen0001_4740	1 kb	Chr5: 8010441–8011481	—	—	GTGTG	—
SweGen0070_3823	7 kb	Chr18: 69245396–69252178	—	—	NA	NA
SweGen0010_5023	1 kb	Chr6: 93117562–93118589	—	*L1*	—	—
SweGen0010_4395	9 kb	Chr8: 128399797–128409200	*L2*	—	—	CC
SweGen0093_2495	22 kb	Chr5: 12805927–12827074	*ERVL*	ERVL	—	—

Note.—NS, novel sequence.

Next, we used TIDDIT (Eisfeldt et al. 2017) to detect deletions overlapping the 1,065 NS clusters in the 96 individuals aligned to PT4. And, the UCSC liftover tool (Hinrichs et al. 2006) was used to confirm that the deleted sequences are not present in GRCh38, thereby confirming that the chimpanzee reference allele (CRA) represent NS relative to GRCh38.

Through this approach, we found 397 deletions overlapping any of the 1,065 clusters of NS aligning uniquely to the chimpanzee genome. We estimate the allele frequency of these NSs by counting the number of individuals carrying the CRA (i.e., heterozygous, or no deletion). Interestingly, we find that the CRA is common (AF > 5%) in 391 of these deletion events. The CRA is present in all 96 individuals in 37 events. Two of these events were manually inspected, revealing that the deletion calls are of high quality, and that the CRA overlapped by those deletions truly are widespread (supplementary figs. S11 and S12, Supplementary Material online). Notably, the deletions are significantly larger than the NS contigs (*P* < 0.00001) in aggregate the 397 deletions cover 1.3 Mb of sequence, and the largest deletion spans 102 kb (supplementary fig. S13, Supplementary Material online). The five largest deletion calls were found to be credible through manual inspection, and it was found that the CRA was present throughout the population (supplementary figs. S13–S17, Supplementary Material online).

Lastly, we manually inspected seemingly clustered deletion calls; such clusters may represent hotspots of deletion events, or complex deletion events. Two complex deletion events were found through this search. The first complex rearrangement consists of two deletions, flanking a roughly 1-kb copy number neutral fragment (supplementary fig. S18, Supplementary Material online). In the second complex event, we find a tandem duplication neighboring a deletion (supplementary fig. S19, Supplementary Material online). We find that these NS events are present throughout the SweGen population and that these complex deletions represent the GRCh38 reference allele.

### Mapping of the Thousand Genomes Data

**Fig. 5.**
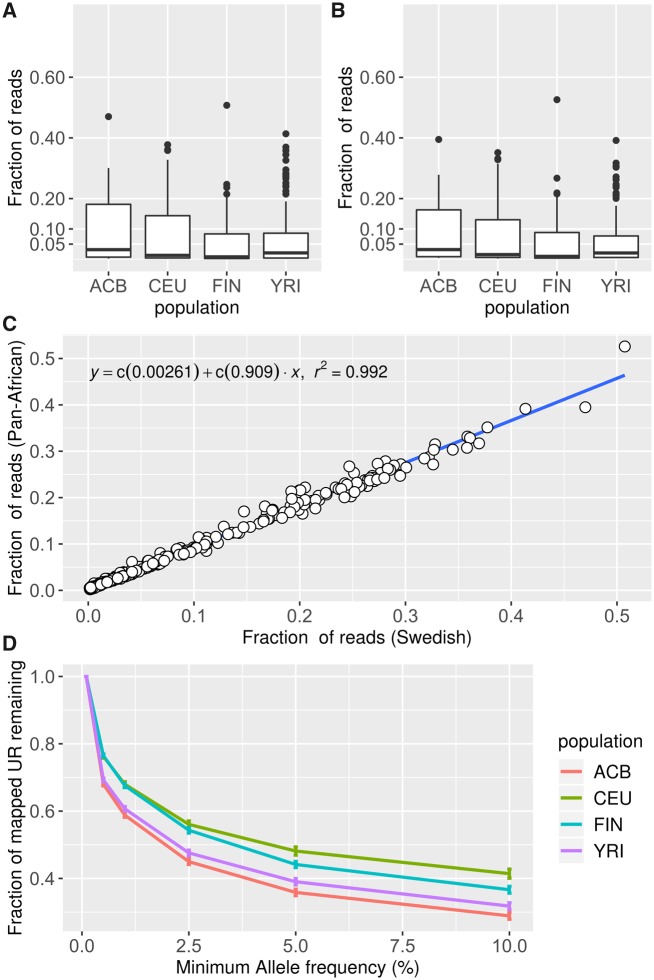
The fraction of URs that align to the (*A*) Swedish NS clusters and (*B*) Pan-African catalog of NS (Sherman et al. 2019). The boxplots are grouped according to the ethnicity of the individuals: African Caribbean from Barbados (ACB), Utah residents with western or northern European ancestry (CEU), Finnish in Finland (FIN), and Yoruba in Ibadan (YRI). The lower and upper hinges correspond to the first and third quartiles of each population, the per population median is indicated by the black horizontal line within the box. (*C*) Scatter plot of the fraction of reads aligning to the Swedish NS and the fraction of reads aligning to the Pan-African NS; each dot represents one of the 1KGP individuals. (*D*) Line plots showing the fraction of aligned UR remaining after removing Swedish NS clusters less frequent than the frequency thresholds (0.5%, 1%, 2.5%, 5%, 7.5%, and 10%); each line represents one of the four 1KGP populations and the error bars indicate the standard deviation among the populations.

The unmappable reads (URs) from four 1KGP populations were aligned to the Swedish NS clusters, as well as a recently published catalog of Pan-African NS (Sherman et al. 2019) (supplementary table S5, Supplementary Material online). On average, 7.4% and 7% of the URs align to the Swedish NS (fig. 5*A*) and Pan-African NS (fig. 5*B*), respectively. Applying a Mann–Whitney *U* test, we found no significant difference in the fraction of UR aligns to the Pan-African NS catalog compared with the Swedish NS (*P* = 0.089); notably, the Pan-African NS catalog is generated from a slightly smaller number of individuals (910) but consists of roughly six times the amount of sequence (296 Mb) compared with our Swedish NS, which is created from 1,000 individuals, but contain only 46 Mb of NS.

There’s a significant overlap between the Pan-African and Swedish NS: 32,794 of the Swedish NS aligns confidently to the Pan-African NS (identity >95%; coverage >90%) (fig. 3*A*). This is also made clear when mapping the UR to the two NS catalogs: the two sequence catalogs correlate strongly, and there’s a linear relationship of the percentages of aligned UR (fig. 5*C*).

We applied ANOVA to test for differences between the populations. First, we tested for differences between the four populations using the Swedish NS as reference; and found no significant differences among the populations (*P* = 0.12). The same test was applied using the Pan-African NS as a reference, which also yielded no statistically significant results (*P* = 0.18).

Lastly, we filtered the SweGen NS based on allele frequency, thereby creating five subsets of the SweGen NS catalog. These partly overlapping subsets consist of NS having minimum allele frequencies of 0.5%, 1%, 2.5%, 5%, and 10%. The URs of the four 1KGP populations were aligned to these subsets of the Swedish NS (fig. 5*D*) revealing that the amount of mapped UR decreases as the rare NSs are removed. On average, 42% of the UR mapping to the Swedish NS align to common NS (allele frequency >= 5%), which is interesting considering that 91% of the Swedish NSs are rare (allele frequency < 5%) (fig. 1*B*). The relative decrease of mapped UR differs across the populations, the CEU has the slowest rate of decrease as rare NS are removed, followed by the FIN population. The URs of the ACB are depleted at a greater rate, and only 29% of the ACB URs mapping to Swedish NSs align to NS more frequent than 10% (compared with 41% of CEU UR). Performing ANOVA and Tukey’s test, we find significant differences between the European (CEU, FIN) and African (ACB, YRI) populations regarding the relative depletion of mapped UR across all frequency thresholds (table 5). Additionally, we find that the FIN and CEU population differ significantly when applying frequency thresholds 5% and 10% (table 5).

**Table 5.** Cross Population Comparison of the Fraction of Remaining Mapped UR When Filtering the Swedish NS Based on Minimum Allele Frequency.

Min AF (%)	ANOVA F Statistica	ANOVA *P* Value	Tukey *P* Values					
			ACB v CEU	ACB v FIN	ACB v YRI	CEU v FIN	CEU v YRI	FIN v YRI
0.5	75.2	<1.1 × 10^−16^	<0.001	<0.001	0.31	0.9	<0.001	<0.001
1	51.4	<1.1 × 10^−16^	<0.001	<0.001	0.16	0.9	<0.001	<0.001
2.5	35.1	<1.1 × 10^−16^	<0.001	<0.001	0.15	0.51	<0.001	<0.001
5	27.9	2.2 × 10^−16^	<0.001	<0.001	0.12	0.032	<0.001	<0.001
10	24.3	1.8 × 10^−4^	<0.001	<0.001	0.25	0.014	<0.001	0.009

Note.—Min AF, minimum allele frequency.

## Discussion

Utilizing the Assemblatron de novo assembly workflow on the 1,000 individuals of the SweGen cohort, we identified 46 Mb of sequence (61,044 distinct clusters), missing from GRCh38 and GRCh37. Each individual genome harbor on average 0.6-Mb unmappable sequence, and there exists a great variety between individuals (fig. 1*B*). Other large scale short read WGS studies, has indicated that NSs are diverse and abundant; these studies include the Simons genome diversity project (average of 5-Mb unmappable sequence/individual) (Mallick et al. 2016) and a recent study of 910 Pan-African genomes (Sherman et al. 2019); additionally, recent studies have confirmed the presence of NS using long read sequencing (Ameur et al. 2018; Audano et al. 2019).

Notably, we report a smaller amount of NS compared with previous studies such as Mallick et al. (2016) and Sherman et al. (2019). We note that Mallick et al. (2016) use the GRCh37 reference genome and do not describe any filtering procedure. The 5 Mb of NS per individual reported in Mallick et al. (2016) is therefore comparable to our 7.8 Mb of unfiltered sequence unmappable to GRCh37 (table 1). Sherman et al. (2019) study a subset of the Pan-African population, which is highly diverse and not as well represented in the current reference genome; additionally, Sherman et al. (2019) utilize a different assembly workflow, involving the extraction and assembly of unmapped reads (instead we perform whole genome de novo assembly), and scaffolding using unmapped read pairs. These differences are therefore due to both technical and biological reasons, the true amount of NS can only be determined using long read sequencing, allowing of the sequencing of the entirety of these NSs.

A great diversity of shared NS was found by aligning the unmappable sequences to the chimpanzee genome, a catalog of Icelandic NS, as well a Pan-African collection of NS (fig. 3*A*). Overall, we find that 70% of the NS align confidently to any of these data sets, and that many of the NS align to multiple data sets. Given the large geographical spread of these data sets, as well as the high similarity between the Swedish NS and those of the public data sets, a significant proportion of NS may be of ancestral origin, and therefore shared not only with the Pan-African population and the chimpanzee, but with other populations as well (fig. 5*A*). We attempted to position the NS using two separate methods: RetroSeq, and alignment to the chimpanzee genome. Overall, we find that both these methods perform relatively poorly: only 246 NSs were confidently positioned across GRCh37 using RetroSeq, greater numbers of NSs were aligned to the chimpanzee genome, however the NS that did align consists largely of LINEs and unmasked sequence, which is atypical to the NS at large (fig. 2*B*). Nevertheless, we find that these two approaches are concordant, and that there is an enrichment of NS within repeat-masked regions, as well as depletion of NS within genes (tables 2 and 3). Through the RetroSeq analysis, we find that there’s a depletion of NS within centromere, however, these results are unreliable due to the low mappability within such regions. The RetroSeq analysis detected 2,195 NS insertions within 2,384 genes, 245 of these genes are MIM morbid genes. Similarly, through alignment to the chimpanzee genome, we find 2,807 NSs positioned within 143 human gene orthologs. We find that NSs tend to cluster into hotspot regions within some genes, in other genes, we find that the NSs are more dispersed, and yet in other genes, we find only a few, or even a single NS (supplementary tables S2 and S3, Supplementary Material online): illustrating that the NS may form through various genetic mechanisms. Since NSs aligning to PT4 are likely to represent ancestral deletion events, we performed a manually inspection of the BPJs of ten such events. We found four ancestral deletions that were flanked by matched repeat elements, including *Alu*, L1, and ERVL (table 4) indicating that those events were formed through nonallelic homologous recombination between the matched repeats (Lupski 2015). In one ancestral deletion, no repeat elements was present in the BPJ, but a five nucleotide stretch of microhomology was present indicating microhomology-mediated end joining (table 4) (Lupski 2015). Finally, in four of the ancestral deletions the BPJs were blunt indicative of nonhomologous end joining (Lupski 2015; Nazaryan-Petersen et al. 2018). In one case, we were unable to find any reads spanning the ancestral deletion (SweGen0070_3823). Since the breakpoints of this NS are located in low-mappability regions, one plausible explanation is that those regions are in fact chimpanzee segmental duplications. However, we cannot confirm this hypothesis since no such tracks available in PT4. In addition, when we searched all NS mapping to PT4 we found two clustered ancestral deletions highly suggestive of underlying complex rearrangements (Nazaryan-Petersen et al. 2018). This shows that a small proportion of NS may originate from ancient complex deletion events (supplementary figs. S18 and S19, Supplementary Material online).

Overall, there’s clearly an abundance of NS within genes; previous studies has shown that NS inserted within genes may affect gene regulation (Kehr et al. 2017), highlighting the importance of studying NS in rare disease research. Notably, the RetroSeq and contig alignment approaches differ: aligning the contigs directly to the chimpanzee genome we find that many contig clusters align closely within the same region (fig. 4*A* and *B*), instead, RetroSeq tend to position a single contig on multiple positions. This difference is likely due to the similarity of the contigs, and that the signal (i.e., read pairs) is likely to be distributed randomly across these similar contigs—producing either low support calls or hybrid calls that are not considered in our analyses. Additionally, we apply an allele frequency cut off (a minimum of five individuals) to the RetroSeq calls. Finally, we find that the two methods produce different sets of genes, which may also explain the observed differences (supplementary tables S2 and S3, Supplementary Material online). Overall, it is clear that long read sequencing would be necessary not only to position the NS but also to prevent similar NS from being collapsed into a single contig.

We aligned the WGS data of 96 individuals to PT4 and performed structural variant (SV) calling using TIDDIT. Through that analysis, we noticed that the NSs are fragmented. Compared with our de novo assembly approach, we discover nearly three times as much NS by characterizing the deletion events in the GRCh38 reference (supplementary table S4, Supplementary Material online). Further, we find that many of the ancestral NSs are common (supplementary table S4, Supplementary Material online), which stands in disagreement to the results shown in figure 1. These discrepancies are likely due to the fragmentation of the NS, causing similar NS to appear different due to various technical artifacts, including low coverage across heterozygous sequences, and the formation and popping of bubbles at otherwise homozygous NS. We note that other previous studies report a large amount singleton NS (Sherman et al. 2019), indicating that these problems are widespread.

In aggregate, these findings show that the ancestral NS may be more homogenous than previously thought, and that the allele frequencies of polymorphic ancestral sequence are underestimated.

Our findings here show that there is great benefit in expanding the reference genome. However, due to the large amount of low frequency sequences, the expanded reference would quickly become very large and complex. In 1,000 individuals from a single population, we report 46 Mb of unmappable sequence, which is comparable in to the length of chromosome 21, which is still small compared with the nearly 300 Mb across 910 Pan-African individuals. In addition, it is well known that many unmappable sequences are repetitive and/or related to various mobile elements (Ameur et al. 2018), here, we show that the NS are enriched by satellites and simple repeats (fig. 2*B*), further complicating the alignment and analysis of these sequences.

These difficulties would likely be overcome through the use of graph reference data structures (Paten et al. 2017), combined with long read or linked read sequencing. Although, a more diverse reference genome is needed, we do find that the NSs differ greatly among populations and individuals (fig. 5*A* and *B*), most of the NS are rare, and each sequenced individual adds significant amounts of new NS (fig. 1*B*) (Sherman et al. 2019). Using our NS catalog as a reference, and comparing the results to that of the Pan-African NS, we find that both perform relatively poorly as references for unmappable sequence: Both NS catalogs absorb roughly 7% of the UR, additionally we find that the Swedish and Pan-African NSs correlate strongly (fig. 5*C*), indicating that these individuals share a significant amount of common NS (fig. 3*A*). On the other hand, we find that 18,773 sequences could be confidently aligned to PT4 but not to GRCh38 (compared with 32,794 sequences confidently aligned to the Pan-African NS, but not GRCh38). Considering that PT4 is constructed from 6× WGS of a single individual (https://www.ncbi.nlm.nih.gov/assembly/GCF_000001515.5/; last accessed August 12, 2019), it may be cost efficient to add chimpanzee specific sequences to a human graph genome. Similar ideas are explored in Sundaram et al. (2018) where the authors create an annotation tool based on common single nucleotide variant found in nonhuman primates and show that such approach may be useful to assess the pathogenicity of rare variants in the human genome.

In summary, we present 46 Mb of strictly quality controlled NS in 1,000 Swedish genomes, we find that most of our NS contigs are rare; but also, that a substantial amount these NS contigs represent common alleles; indicating that the NS are fragmented. Additionally, we find that these sequences are enrichment in simple repeats and satellites. Due to the repetitive nature of the NS, long read sequencing would be necessary not only to find but also to position the NS. Using the NS of nearly 2,000 individuals, we were able to align only a fraction of the UR of four 1KGP populations, indicating that greater diversity within the reference genome is necessary, but also that it may be more cost efficient to expand the human genome with sequence originating from great apes.

## Materials and Methods

### Whole Genome Sequencing Data

Two data sets were studied: The SweGen cohort (Ameur et al. 2017), WGS data from the 1000 Genomes project (1KGP).

The SweGen cohort consists of WGS data of 1,000 Swedish individuals. Each individual was sequenced to an average of 30× coverage, using a polymerase chain reaction-free 150-bp paired-end library. All samples were prepared from blood. More details on the SweGen data set can be found in the SweGen paper (Ameur et al. 2017), as well as on the Swefreq website (https://swefreq.nbis.se/; last accessed August 12, 2019).

The WGS data from 1KGP was retrieved from the UPPMAX computational infrastructure. WGS data of four populations (African Caribbean from Barbados [ACB], Utah residents with western or northern European ancestry [CEU], Finnish in Finland [FIN], and Yoruba in Ibadan [YRI]) were randomly selected for analysis based on the criterium that each population needed to contain roughly 100 individuals. For more information on the 1KGP data set, visit the UPPMAX and 1KGP websites.

### De Novo Assembly of the SweGen Data Set

The SweGen data set was assembled and analyzed using the default Assemblatron workflow (0.1.0) (https://github.com/J35P312/Assemblatron; last accessed August 12, 2019). Assemblatron performs de novo assembly using an approach similar to FermiKit (Li 2015): Reads are error corrected using BFC, a FM-index is constructed using RopeBWT2, and the de novo assembly is performed using the Fermi2 assembler (Li 2012). Compared with FermiKit, Assemblatron uses KMC (Deorowicz et al. 2015) for k-mer counting, and the BFC-KMC branch for error correction. The Assemblatron workflow is freely available through Github, and may be run using a Singularity container. The Fermi2 workflow was run using the Assemblatron (0.1.0) singularity image:

singularity exec assemblatron.img python /bin/assemblatron.py --assemble --fastq input.fastq --prefix $3 -k 41 --tmp temp

### Analysis of Unmappable Contigs

The contigs were aligned to GRCh37 using the intracontig mode of BWA-MEM (Li 2013), using the Assemblatron BWA-MEM wrapper:

singularity exec assemblatron.img python /bin/assemblatron.py --align --ref ref.fa --prefix contigs.fa --contigs contigs.fa

The contigs that did not align to GRCh37 were extracted using samtools (Li et al. 2009):

samtools view –bh –f 4 input.bam | samtools fastq - > unmapped.fastq

The unmappable contigs were clustered using CD-hit (Li and Godzik 2006) and all unmappable contigs were stored in a single Fasta file, and the name of each sequence was set to the following format:

IndividualXYZ_I

where “IndividualXYZ” is a unique id given to each individual, and I is an id given to each sequence of that individual. The frequency of each sequence was determined using the “.clusters” file produced by CD-hit. This file describes which sequences are present in which cluster. CD-hit was run using the following command:

cd-hit-est -i merged.fasta -o output_clusters -aS 0.90 -aL 0.90 -c 0.95 -B 1 -M 60000 -T 16

Contaminant sequence clusters were removed through alignment to the comprehensive BLAST (Altschul et al. 1990) nucleotide database:

blastn -query output_clusters.fasta -db nt -outfmt “6 qseqid sseqid sgi evalue staxids sskingdoms qstart qend” -num_threads 16 > blast_results.tab

where output_clusters.fasta is the cluster fasta file produced by CD-hit. Clusters having significant matches to nonprimates, but no significant match to primates were removed. A significant match was defined as any alignment having an *E* score < 10^−^^40^. Clusters represented by sequences <300 bp were also removed.

The remaining clusters were aligned to GRCh38, Pan_tro 4.0 (PT4), a catalog of Pan-African sequences (Sherman et al. 2019), and a catalog of Icelandic NSs (Kehr et al. 2017) using NUCmer (Marçais et al. 2018), which was run using the default settings. The resulting alignments were filtered using the show-coords tools of MUMmer:

show-coords -H -T -c in.delta > out.coords

Alignments having a sequence identity >95%, and a coverage >90% were considered confidently aligned, and were used through the downstream analyses. Sequence clusters aligning poorly (sequence identity <80%, and coverage <50%) to the GRCh38.p12 primary assembly were considered to be NS. The GRCh38.p12 primary assembly was downloaded from the GenCode FTP (https://www.gencodegenes.org/human/; last accessed August 12, 2019) and contains autosomes, sex chromosomes, mitochondria, as well as unplaced scaffolds.

### Repeat Enrichment Analysis

The repeat contents of the NS and GRCh37UC were analyzed using the RepeatMasker tool (http://www.repeatmasker.org/; last accessed August 12, 2019), which was run using the default settings. The significance of the enrichment/depletion of repeat classes was analyzed through nonparametric permutation tests. Pairwise tests were performed between sets of sequences. Such tests include the comparison of GRCh38 to the NS as well as comparisons between the NS and GRCh37UC. These two sets are defined as set *A* and set *B*, where set *A* contains at least twice as many contigs than B.

Initially, the absolute value of the percentage difference between each repeat class of set *A* and B was computed:

$${\text{Diff}}_{\text{rep}}=\left|A_{\text{rep}}\%-B_{\text{rep}}\%\right|.$$

Here, *A*_rep_% and *B*_rep_% represent the percentage content of the repeat class “rep” in sets *A* and *B*, respectively, and Diff_rep_ represent the absolute difference between these two percentages. Diff_rep_ was computed for each major repeat class reported by RepeatMasker, including, LINEs, SINEs, RNA, DNA-element, simple repeat, satellites, and unmasked sequence.

Next, a random subset of contigs is chosen from set *A*. This random subset, denoted *R* is chosen so that it contains the same number of contigs as set *B*.

Thereafter, the absolute values of the percentage differences between each repeat class of set *A* and *R* are computed:

$$\text{Diff}\_R_{\text{rep}}=\left|A_{\text{rep}}\%-R_{\text{rep}}\%\right|.$$

Random subsets (10,000) were samples for each comparison presented in this study, and the *P* value is defined as the fraction of times where

$$\text{Diff}\_R_{\text{rep}}>{\text{Diff}}_{\text{rep}}.$$

Throughout these comparisons, GRCh38 was represented as 500,000 random sequences sampled across GRCh38. These 500,000 sequences were uniformly sampled across GRCH38, and the size distribution of these sequences follows the size distribution of the GRCh37UC.

### Positioning of the NSs

The NSs were positioned using RetroSeq (Keane et al. 2013). RetroSeq was run in the alignment mode, and the quality controlled GRCh37UC clusters were provided as input mobile element sequence, using the -eref parameter. The RetroSeq analysis was performed using a custom RetroSeq fork, using BWA-MEM (Li 2013) instead of the standard exonerate (Slater and Birney 2005).

Each of the 1,000 individuals were analyzed using RetroSeq, and the resulting bed files were merged using DBSCAN (Ester et al. 1996), setting the epsilon distance to 150 bp, and the minimum number of neighbors to 3. The DBSCAN algorithm was run so that only insertion events involving the same type of insertion (i.e., NS) was merged, clusters containing insertions from <5 individuals was discarded, and any insertion of GRCh37UC matching GRCh38 were removed; additionally, we removed so called hybrid insertion calls. Briefly, a hybrid insertion call is the insertion of multiple NSs at a single genomic position, or the insertion of a sequence consisting of multiple NSs; such call may be produced due to technical artifacts such as misalignment or fragmented NS, as well as insertion of true heterozygous NS.

### Sequence Feature Enrichment Analysis

The enrichment of various genomic features at the insertion breakpoints (segmental duplications, centromeres, genes, and repeat elements). The positions of genes were downloaded via the ENSMBL Biomart (Zerbino et al. 2018), and the genomic position of segmental duplications, repeat-masked elements, and position of centromeres were downloaded via the UCSC Tablebrowser (Karolchik et al. 2004). The insertion breakpoints were found either through the previously mentioned RetroSeq analysis, or by aligning the NSs to the chimpanzee reference genome PT4.

The intersection of the genomic features and the insertion breakpoints were found using BEDTools intersect (Quinlan and Hall 2010):

Bedtools intersect -wa -wb -a ins.bed -b feature.bed > intersect.bed

Here, ins.bed is the breakpoint position, produced by retroSeq, or by the alignment of NSs using NUCmer. Feature.bed is one of the bed files containing genomic features, and intersect.bed is the output bed file. The command was run multiple times, comparing each set of insertion breakpoints (chimpanzee, and retroSeq) to multiple sequence features.

The statistical significance of the enrichment of various genomic features was found through simulation followed by binomial tests: for 10,000 iterations, n regions of size 1,000 were randomly selected across the genome, and the fraction of regions overlapping a genomic feature (such as genes) was computed, here n is the number of insertions or aligned sequences produced by RetroSeq and NUCmer, respectively. Thereafter, the fraction of observed insertion overlapping the same genomic feature was computed, and a binomial test was performed to test if the observed fraction differed from the simulated one. Such simulations and tests were performed for each combination of genomic feature and set of insertion breakpoints.

### Mapping of the 1KGP WGS Data

The WGS data from the 1KGP individuals was initially mapped to GRCh37, and bam files of URs are available via the 1KGP FTP site. These URs were extracted into one fastq file per individual, and aligned to the GRCh38 primary assembly, using BWA-MEM. Thereafter, the remaining URs were mapped to the previously described CD-hit clusters using BWA-MEM:

bwa mem -p clusters.fasta in.unmappable.fastq | samtools view -bhS - | samtools sort - > out.bam

where clusters.fasta is the output fasta produced by CD-hit, and unmappable.fastq is a fastq file containing the URs; a read was defined as unmappable if it did not align to GRCh38. This command was run separately for every individual.

Lastly, we used samtools to count the number of URs:

N_reads=$( samtools view –bh –F 2048 out.bam | samtools view –F 256 - -c )

as well as to count the number of mapped reads:

N_mapped=$( samtools view –bh –F 2048 | samtools view –bh –F 4 - | samtools view –F 256 - -c )

The same analysis was repeated using the catalog of Pan-African NSs as reference (Sherman et al. 2019).

### Mapping the WGS Reads to the PT4 Reference Genome

Ninety-six individuals were randomly selected from the SweGen data set consisting of 1,000 individuals. The WGS data of these individuals were mapped to PT4 using BWA-MEM:

bwa mem -p -t 16 ref.fa in.fastq | samtools view -bhS - | samtools sort -@ 8 -m 2G - > out.bam

where ref.fa is the PT4 reference genome, in.fastq is the fastq file, and out.bam is the resulting bam file. Next, deletions were detected using TIDDIT 2.7.0 (Eisfeldt et al. 2017), producing one SV variant call file (VCF) per individual:

TIDDIT.py --sv --bam in.bam -o prefix -p 8 -r 8

where in.bam is the input bam file, and prefix is the prefix of the output files. The resulting VCF were merged using SVDB 2.0.0 (Eisfeldt et al. 2017), producing a single frequency annotated VCF:

svdb --build --folder prefix --prefix prefix

svdb --export --overlap 0.7 --db in.db --prefix prefix

where input is a folder containing vcf files, and prefix is the prefix of the output files.

Next, the merged VCF file was filtered for high quality deletion calls using grep, and deletions overlapping the previously assembled NS were found using BEDTools intersect:

grep -E “#|PASS” merged.vcf | grep -E “#|<DEL>” > merged.filtered.vcf

BEDTools intersect -a merged.filtered.vcf -b NS.bed -wa -wb > overlapping_deletions.vcf

where merged.vcf is the merged VCF produced by SVDB, merged.filtered.vcf is a VCF containing only deletions passing the TIDDIT quality filters, and NS.bed is a bed file containing the regions spanned by NS.

Lastly, the UCSC liftover tool (Hinrichs et al. 2006) (https://genome.ucsc.edu/cgi-bin/hgLiftOver; last accessed August 12, 2019) was used to confirm that the deletions cover sequence not present in GRCh38. A deletion was considered to cover sequence not present in GRCh38 if the conversion from PT4 to GRCh38 coordinates resulted in the error message “Deleted in new.”

## Data Availability

The 1,000 Swedish genomes are available upon signing a data access agreement with the Scilifelab data center. The NS catalog is available through the SweFreq website (https://swefreq.nbis.se/; last accessed August 12, 2019).

## Supplementary Material

msz176_Supplementary_Data
