# Supplementary figures and images for: Discovery of Novel Sequences in 1,000 Swedish Genomes
Source: Mol Biol Evol. 2019 Sep 24;37(1):18–30. doi: 10.1093/molbev/msz176 (PMC6984370; doi:10.1093/molbev/msz176)

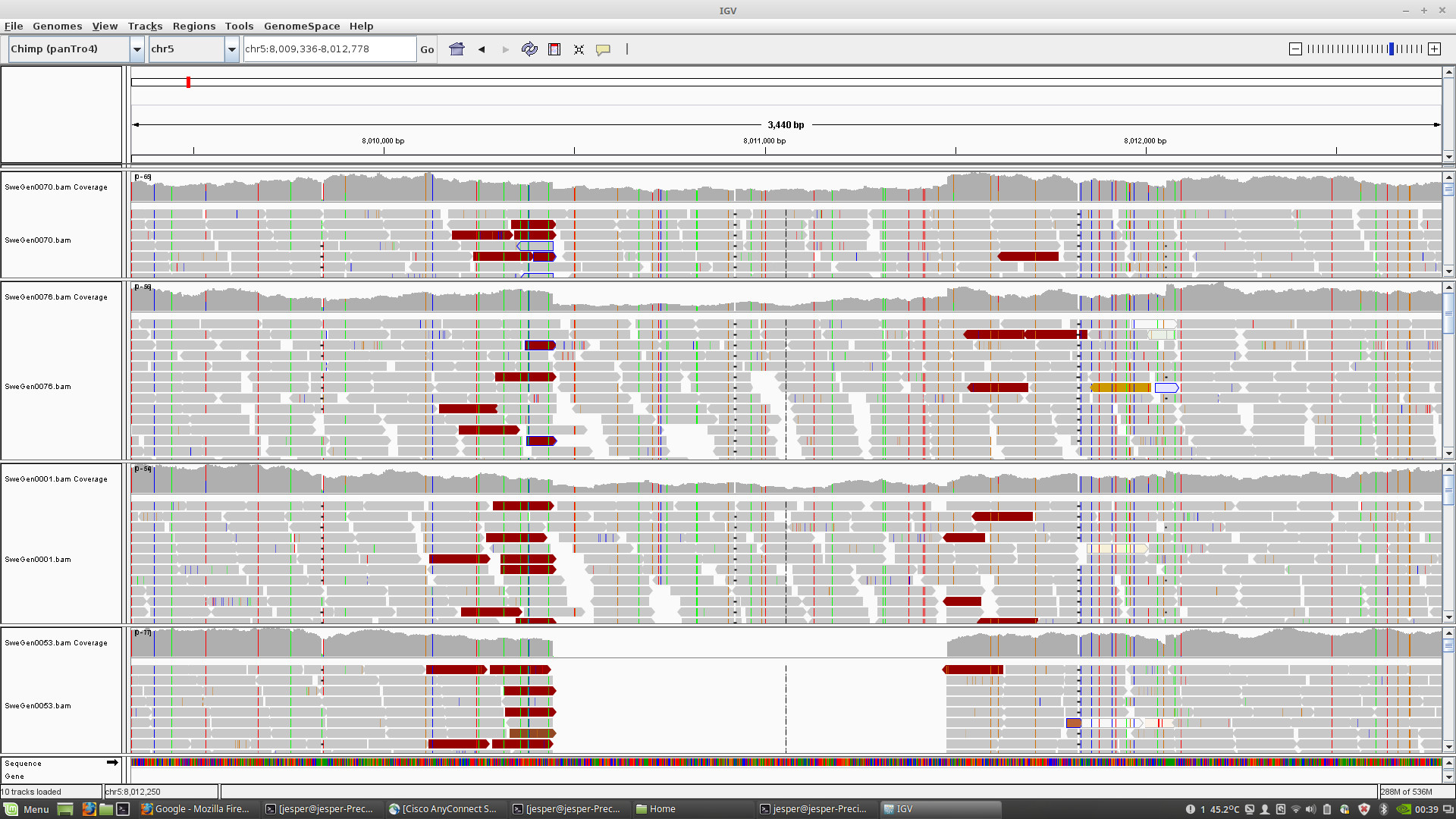

Supplement: msz176_Supplementary_Data [file msz176_supplementary_data.zip › msz176-Suppl_data/Supplementary_Figure S1.jpg]

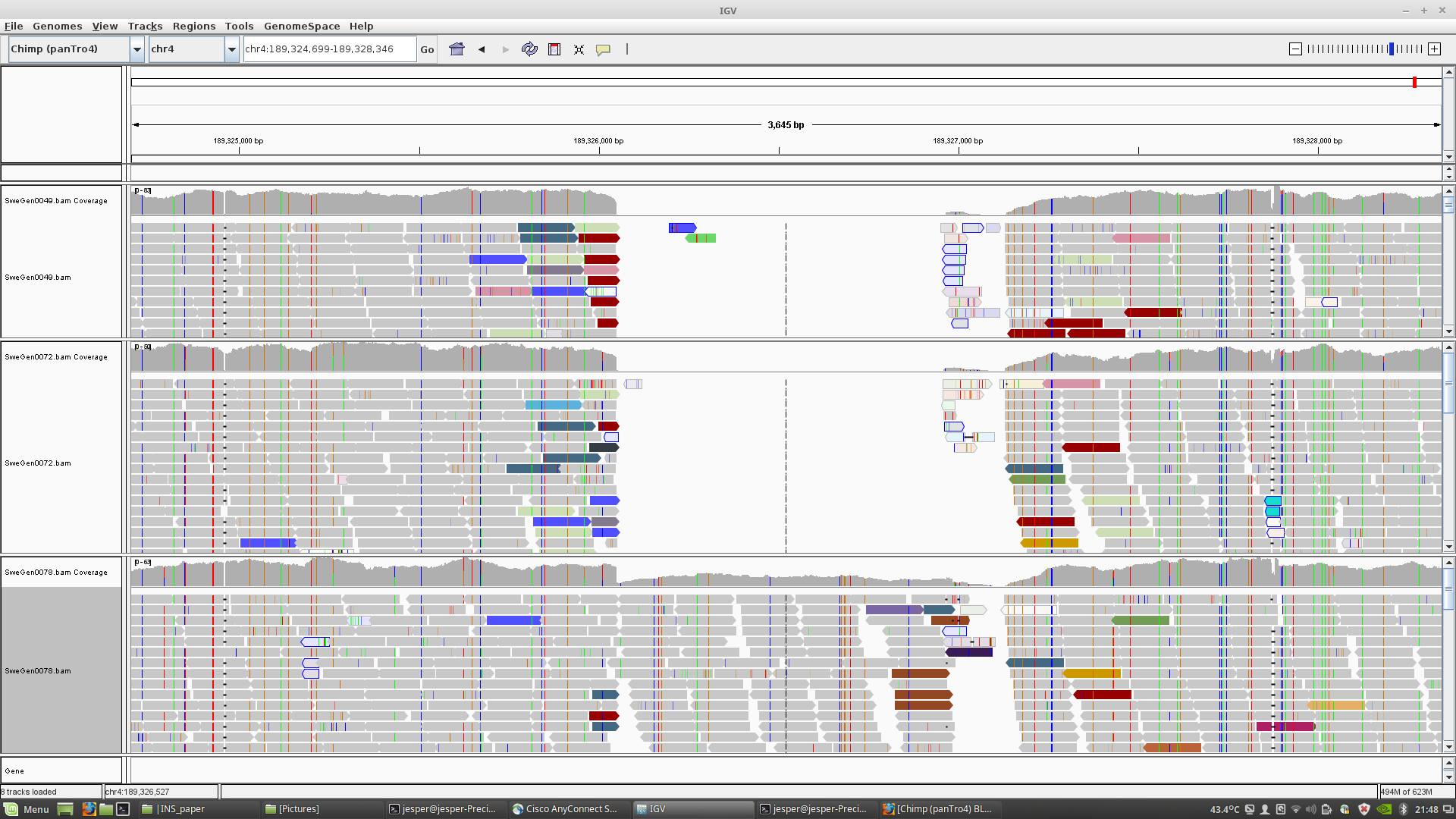

Supplement: msz176_Supplementary_Data [file msz176_supplementary_data.zip › msz176-Suppl_data/Supplementary_Figure S2.jpg]

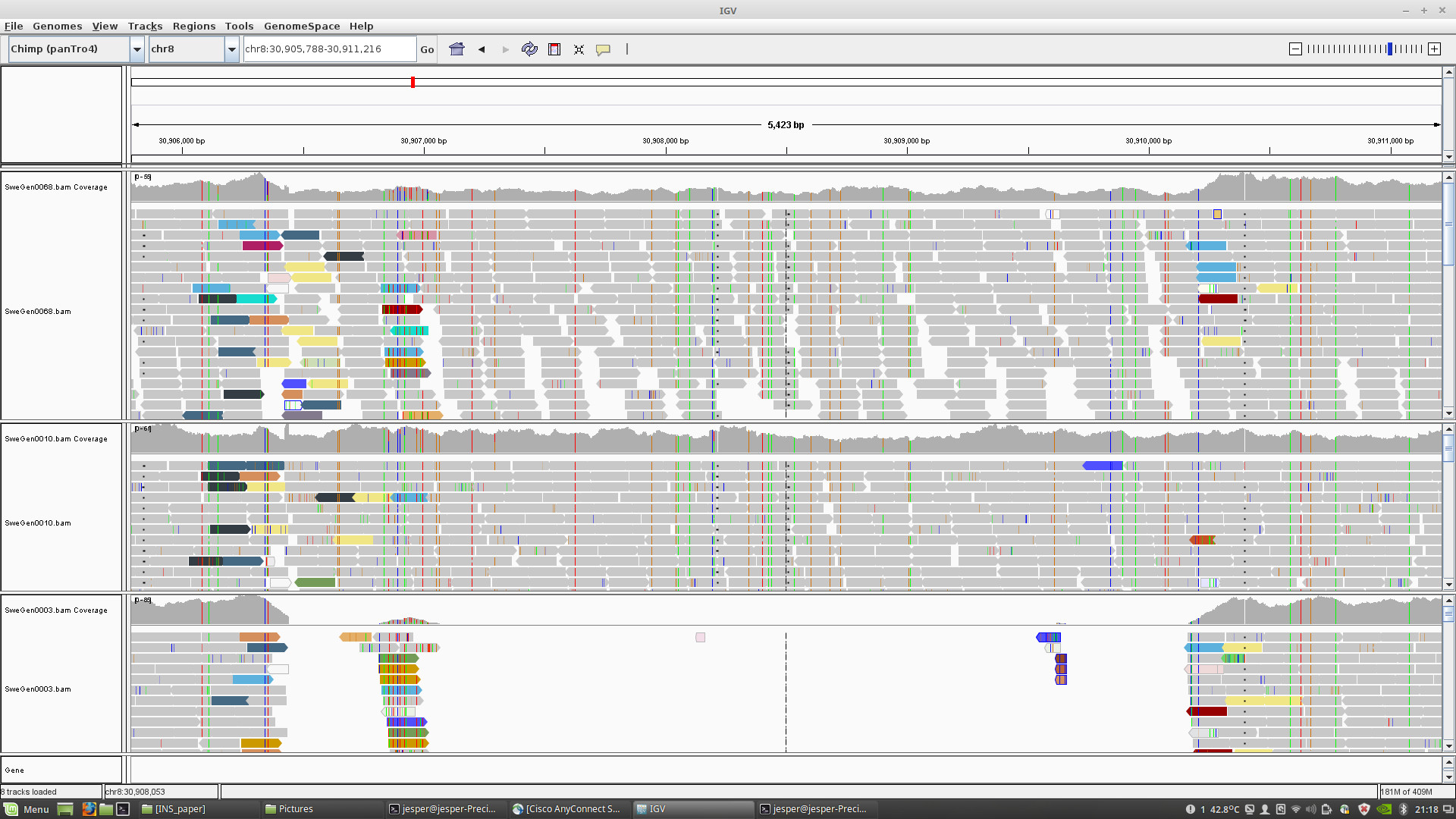

Supplement: msz176_Supplementary_Data [file msz176_supplementary_data.zip › msz176-Suppl_data/Supplementary_FIgure S3.jpg]

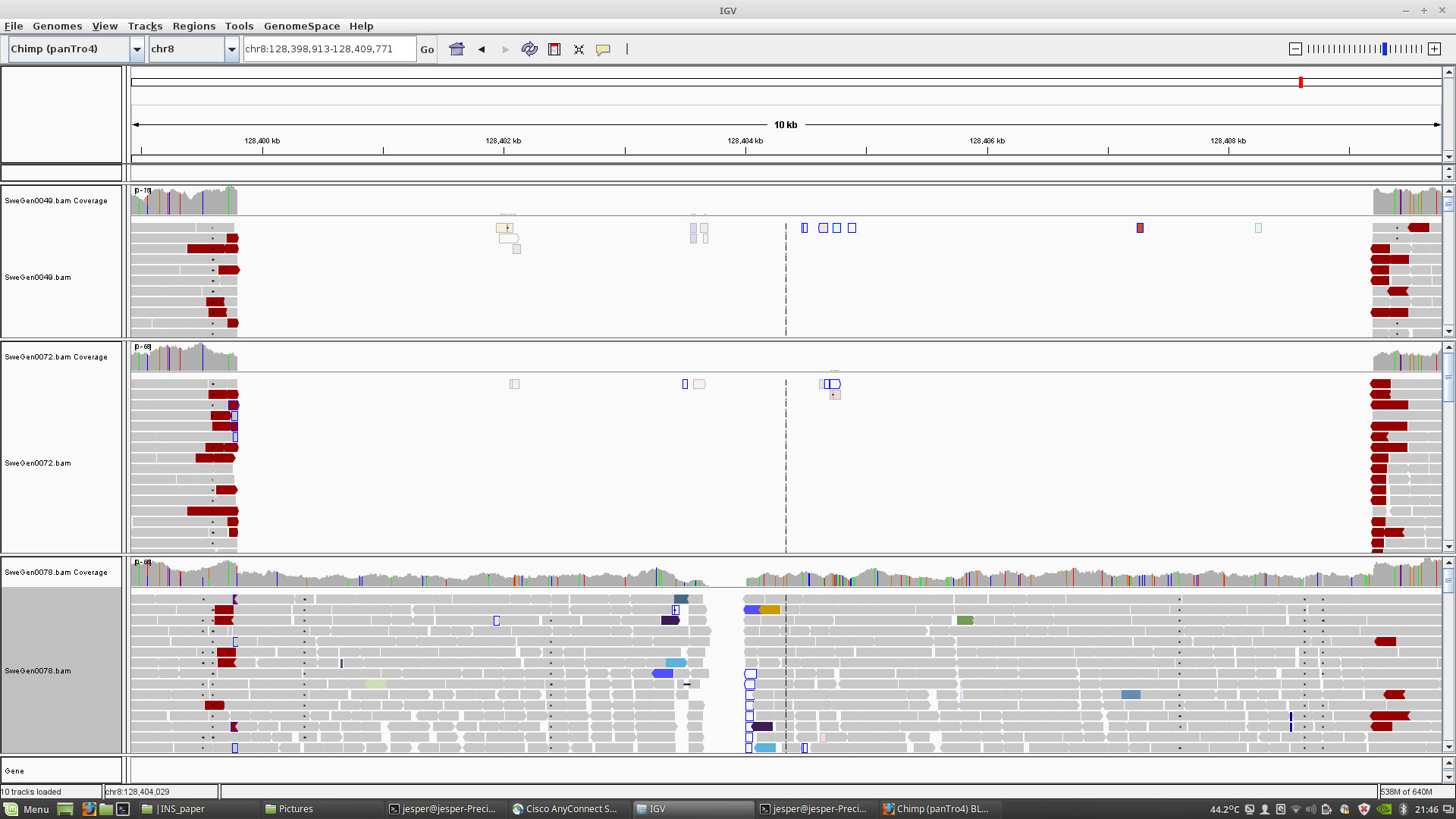

Supplement: msz176_Supplementary_Data [file msz176_supplementary_data.zip › msz176-Suppl_data/Supplementary_Figure S4.jpg]

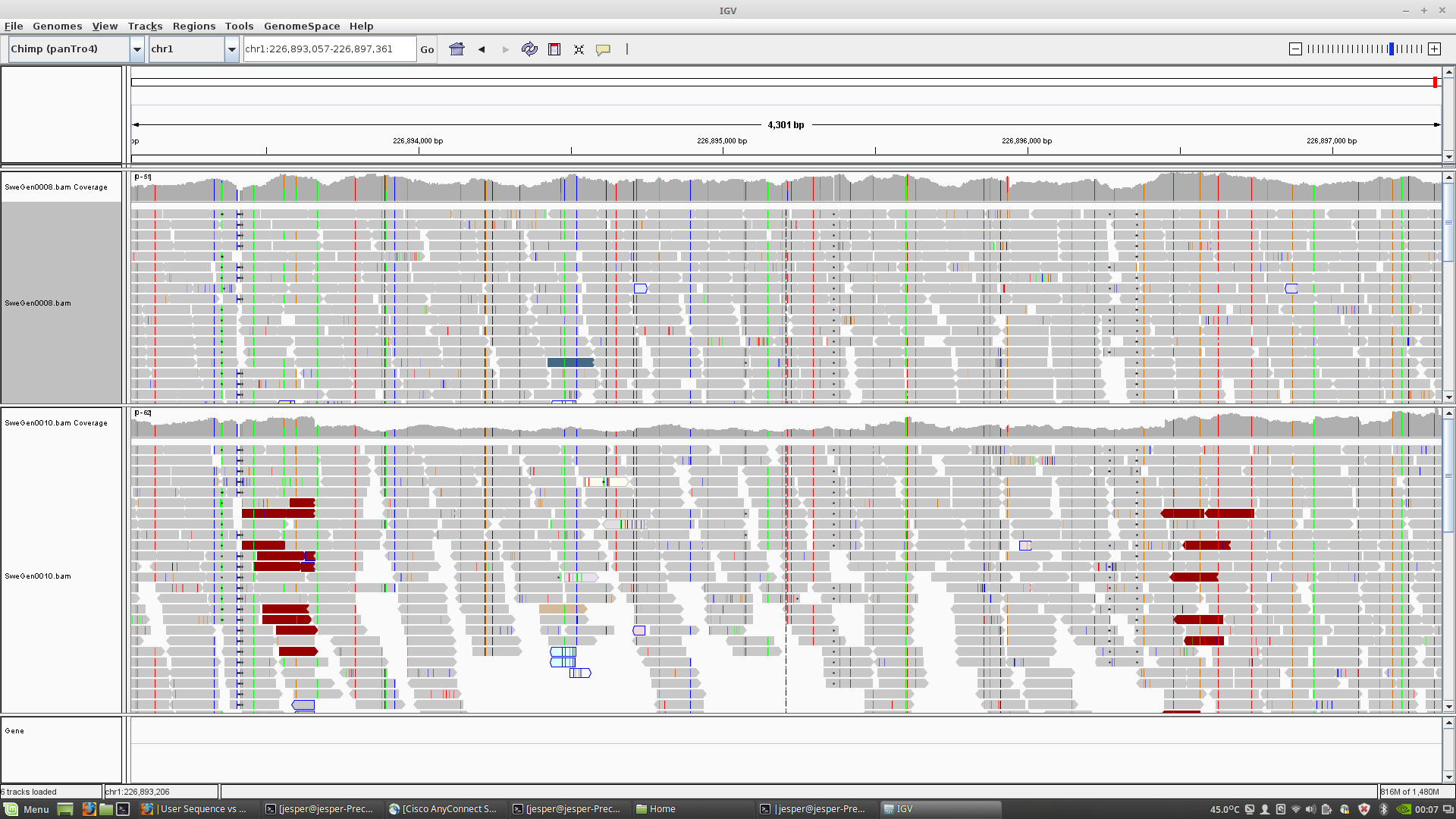

Supplement: msz176_Supplementary_Data [file msz176_supplementary_data.zip › msz176-Suppl_data/Supplementary_FIgure S6.jpg]

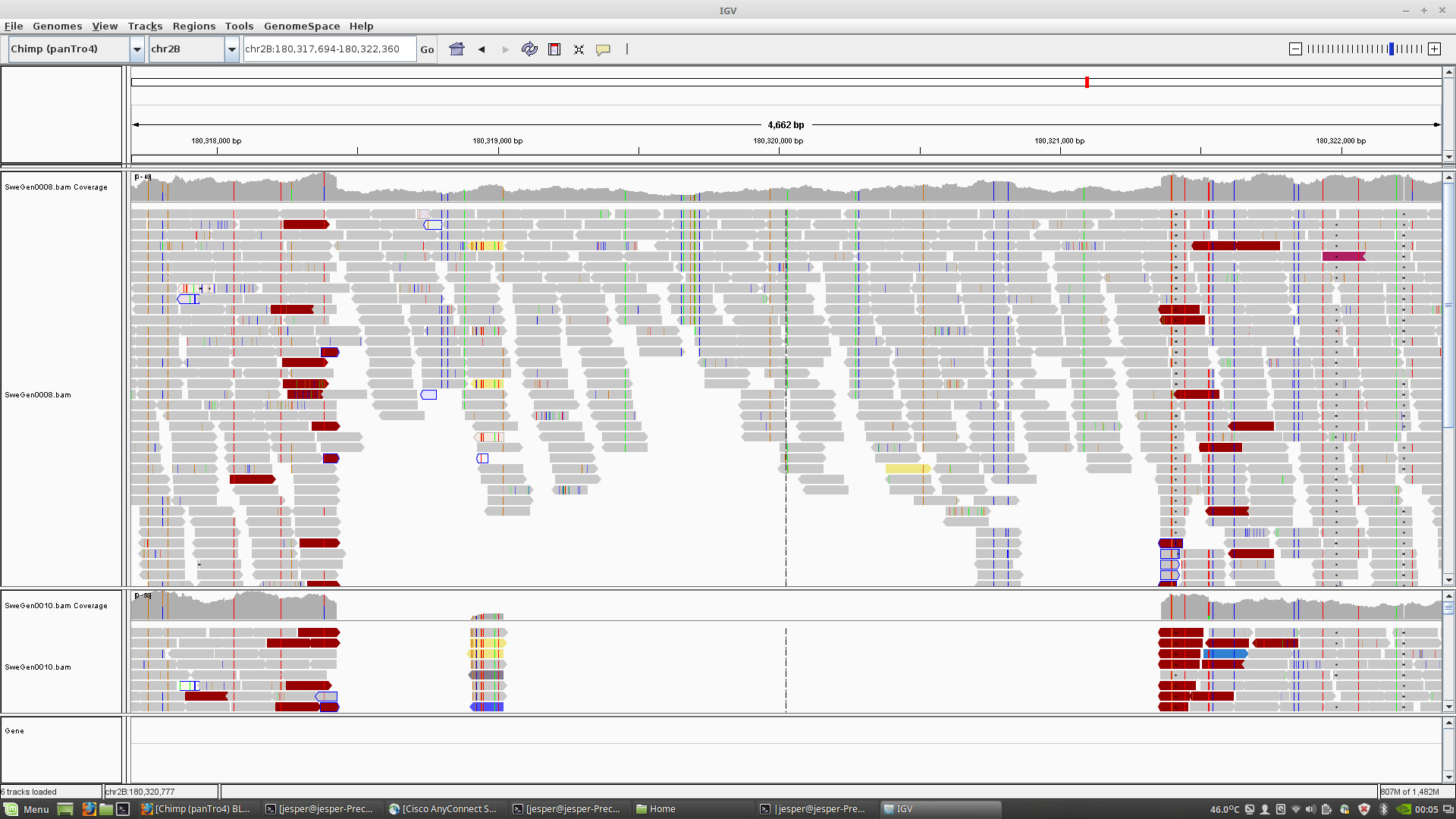

Supplement: msz176_Supplementary_Data [file msz176_supplementary_data.zip › msz176-Suppl_data/Supplementary_FIgure S7.jpg]

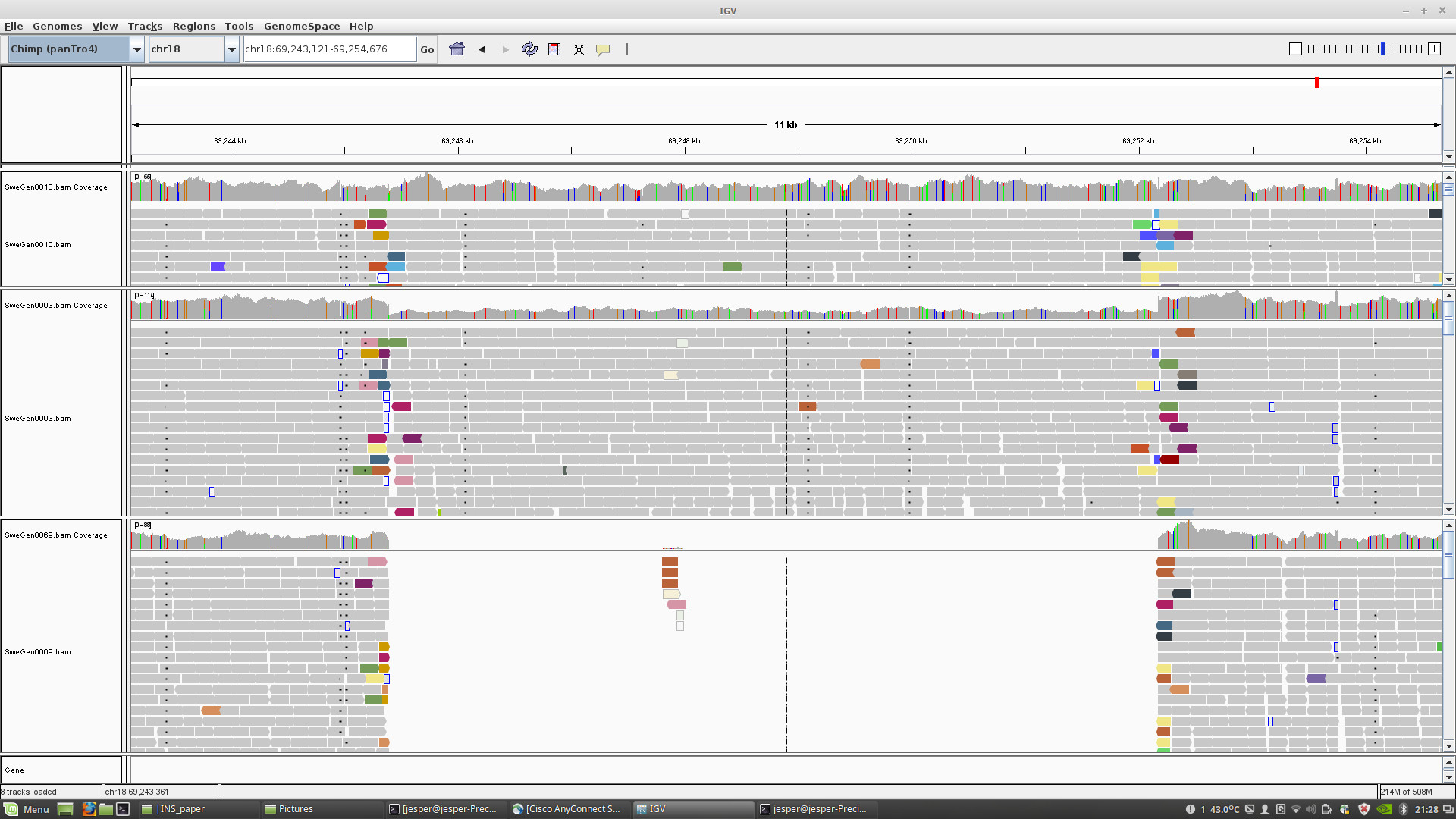

Supplement: msz176_Supplementary_Data [file msz176_supplementary_data.zip › msz176-Suppl_data/Supplementary_FIgure S8.jpg]

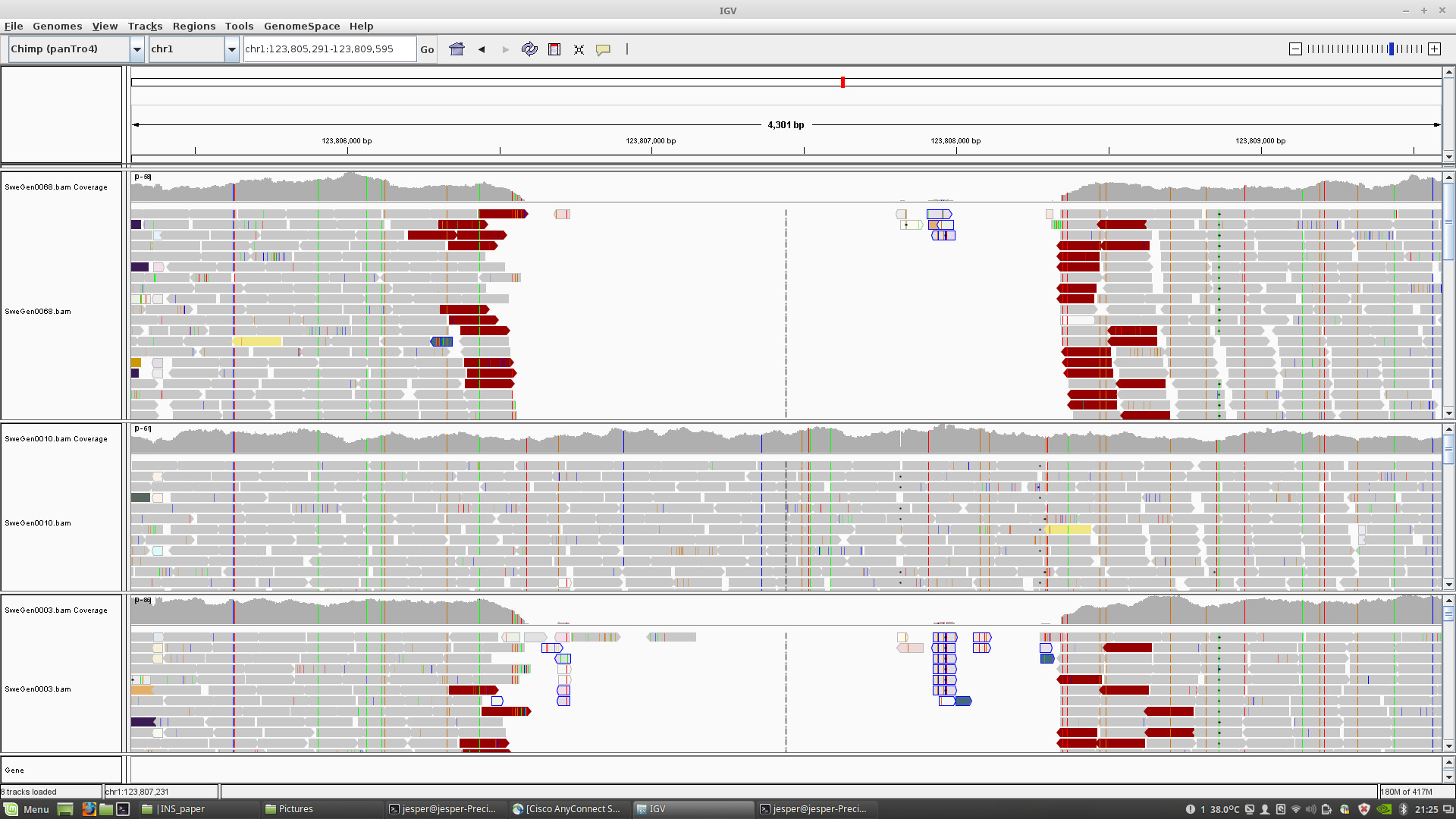

Supplement: msz176_Supplementary_Data [file msz176_supplementary_data.zip › msz176-Suppl_data/Supplementary_FIgure S9.jpg]

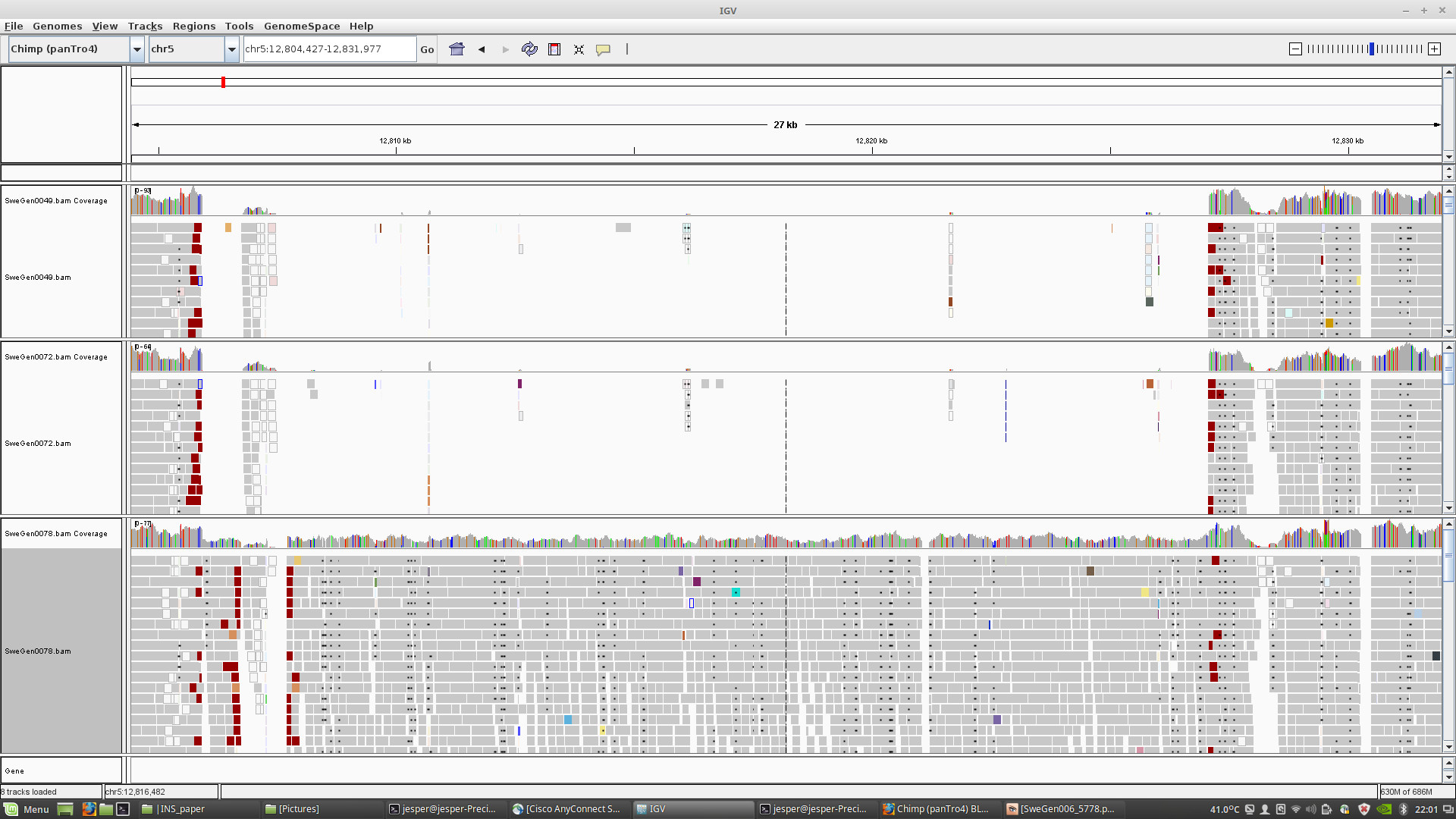

Supplement: msz176_Supplementary_Data [file msz176_supplementary_data.zip › msz176-Suppl_data/Supplementary_Figure S10.jpg]

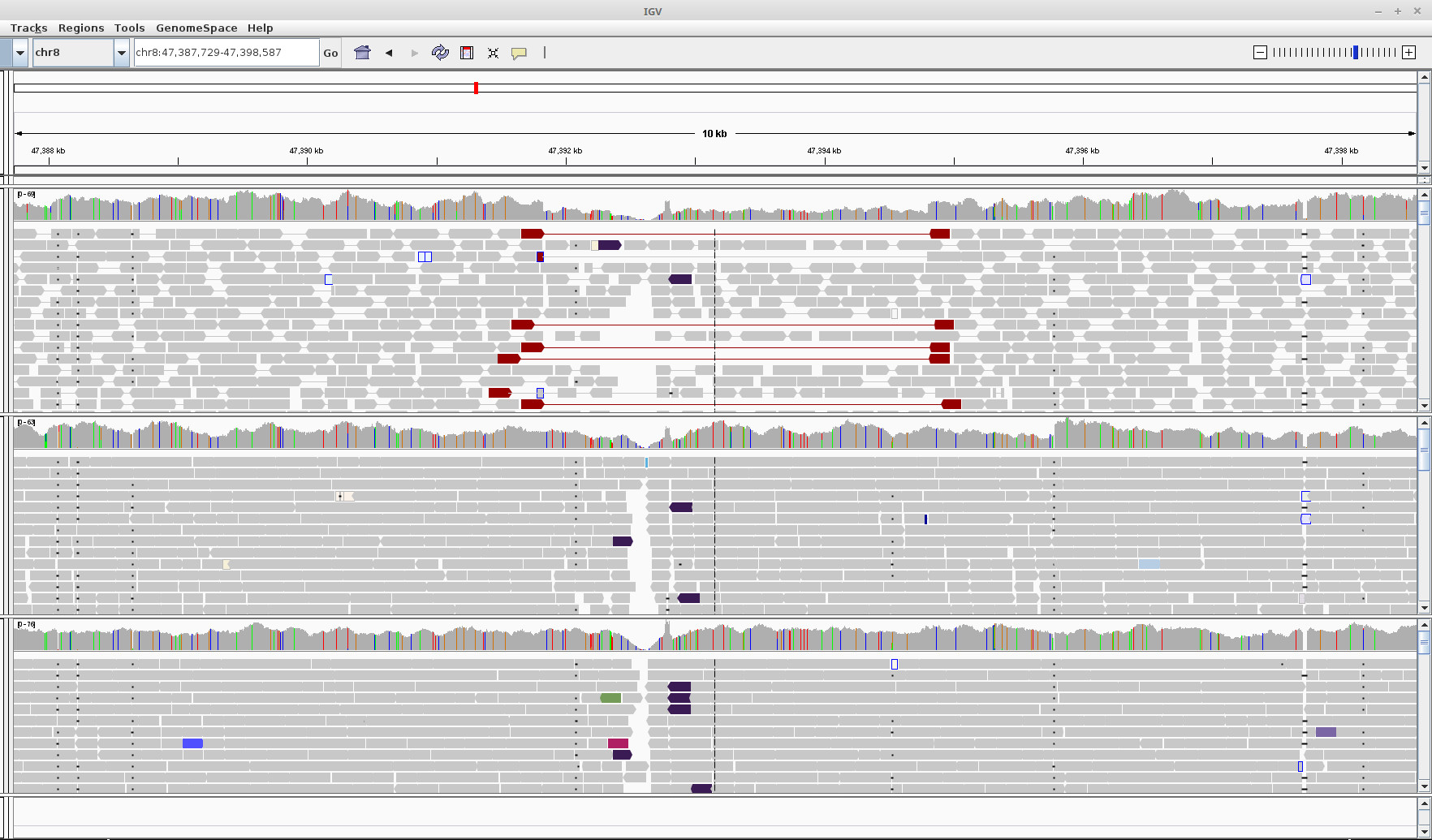

Supplement: msz176_Supplementary_Data [file msz176_supplementary_data.zip › msz176-Suppl_data/Supplementary_Figure S12.jpg]

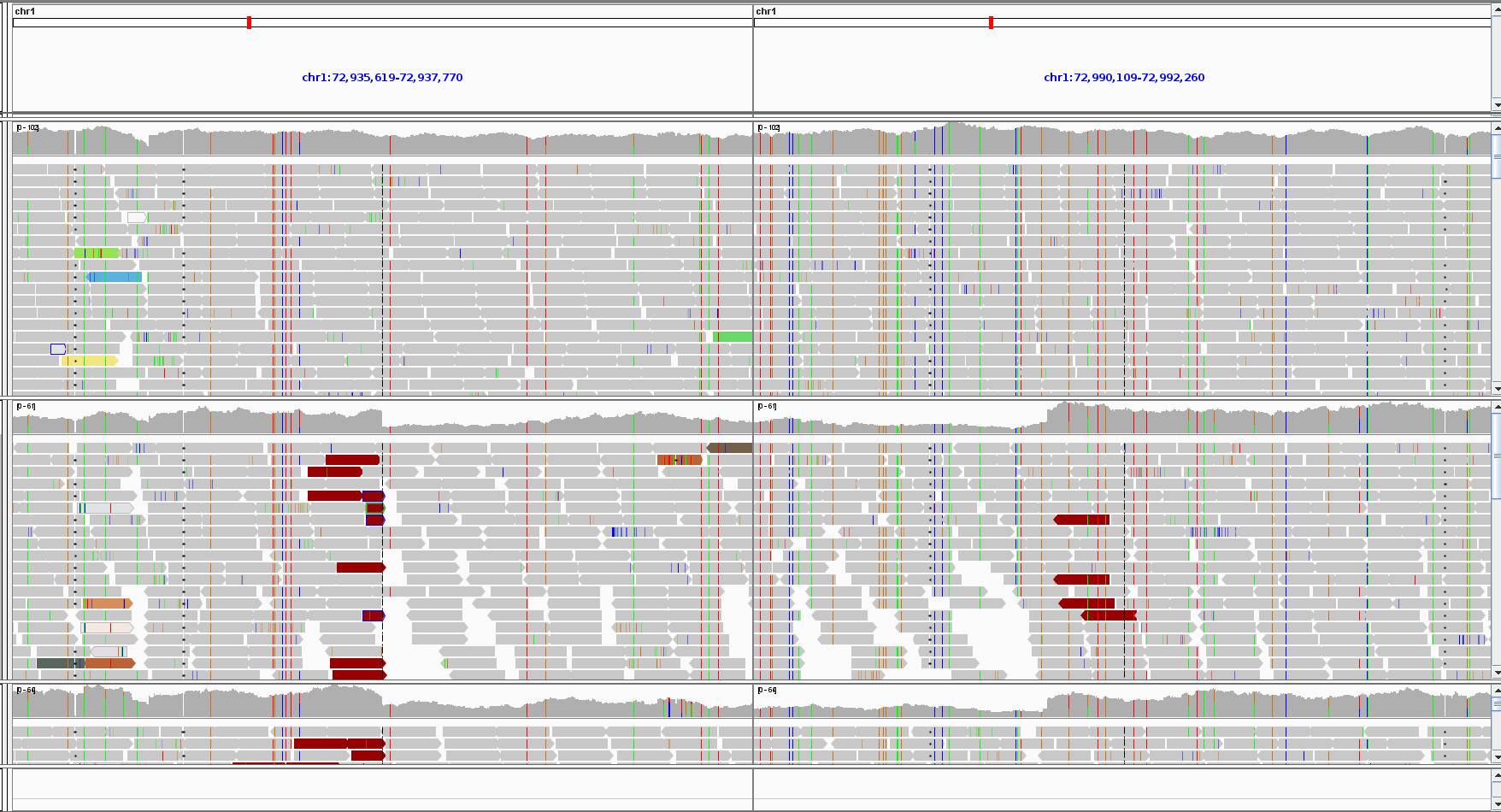

Supplement: msz176_Supplementary_Data [file msz176_supplementary_data.zip › msz176-Suppl_data/Supplementary_Figure S14.jpg]

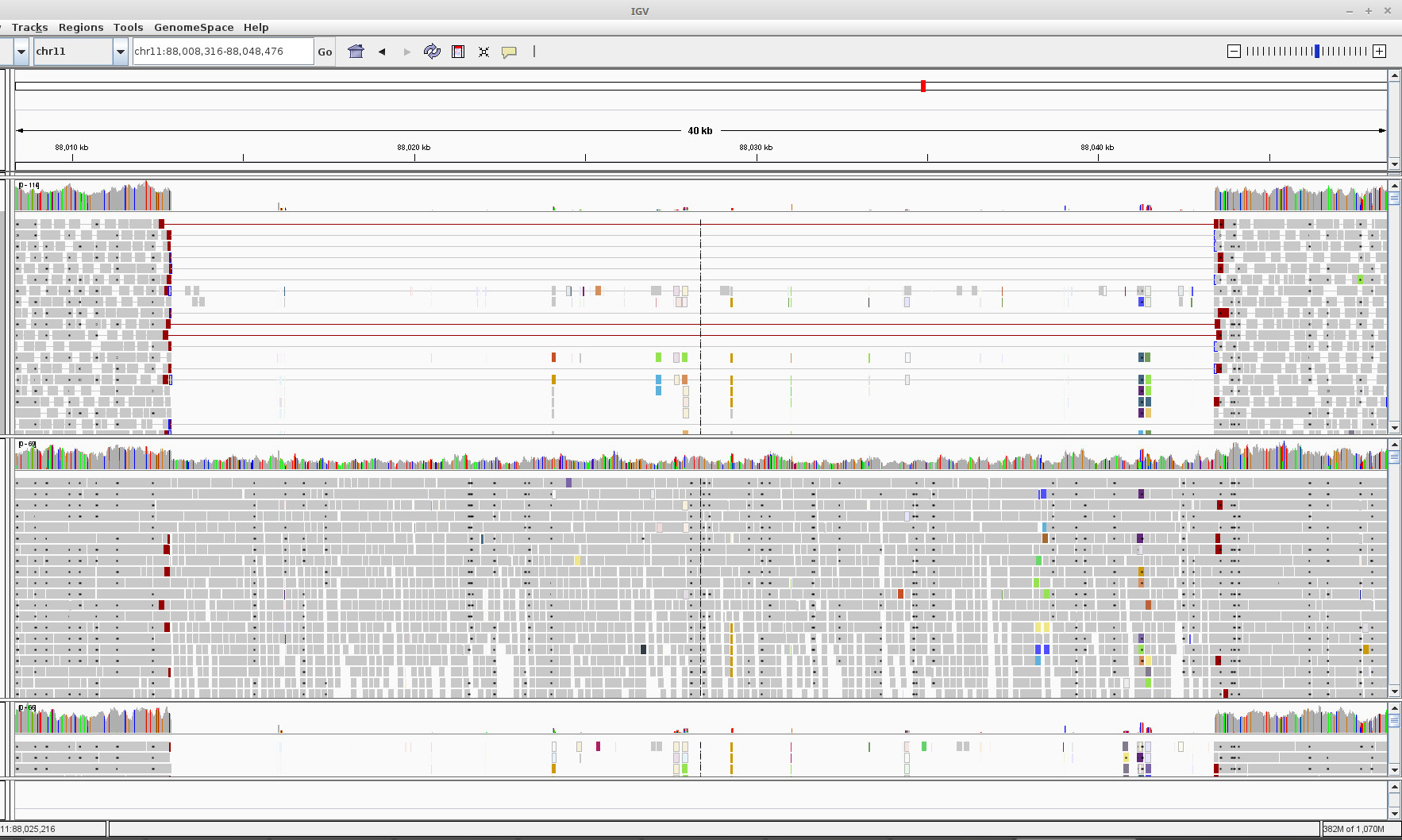

Supplement: msz176_Supplementary_Data [file msz176_supplementary_data.zip › msz176-Suppl_data/Supplementary_Figure S15.jpg]

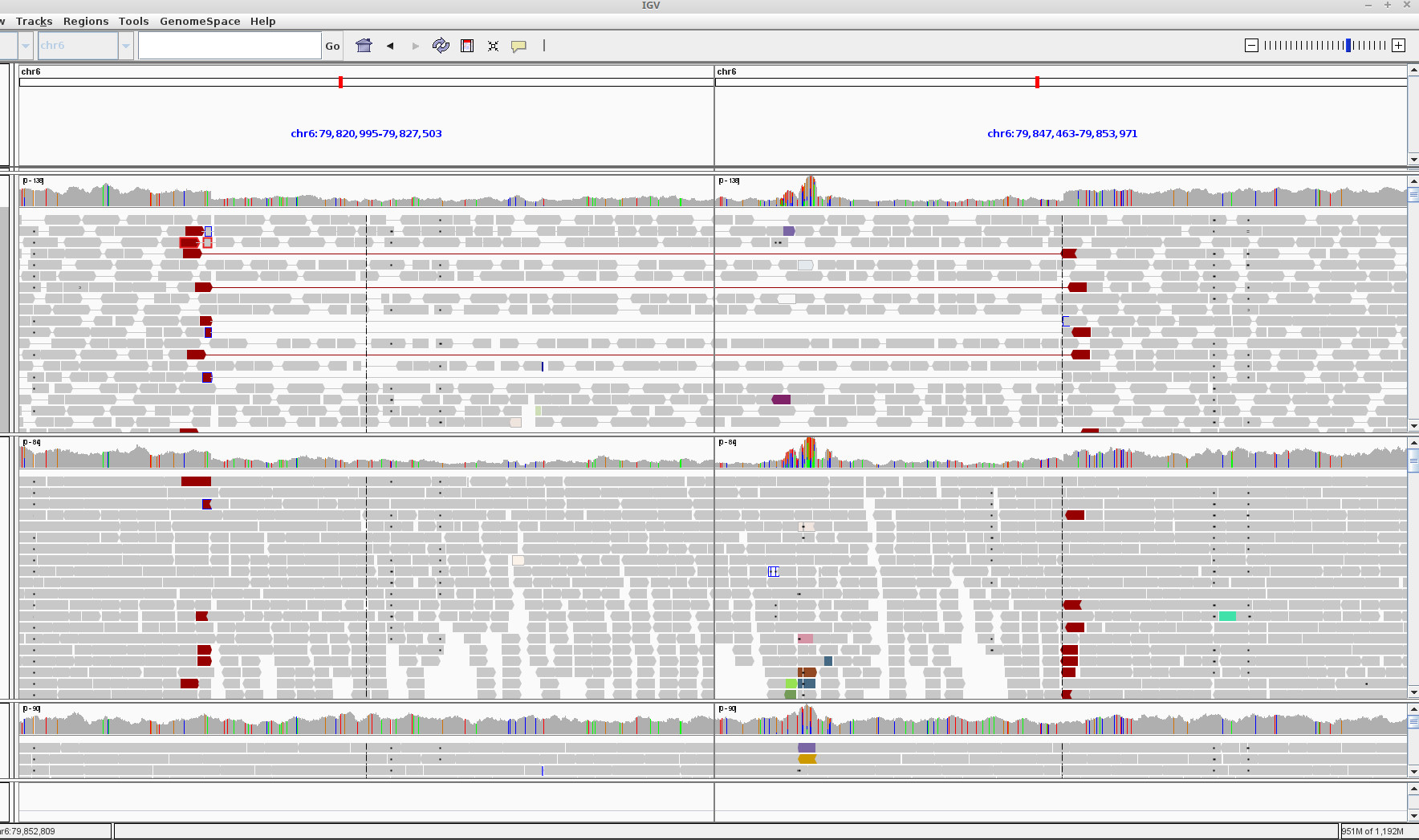

Supplement: msz176_Supplementary_Data [file msz176_supplementary_data.zip › msz176-Suppl_data/Supplementary_Figure S16.jpg]

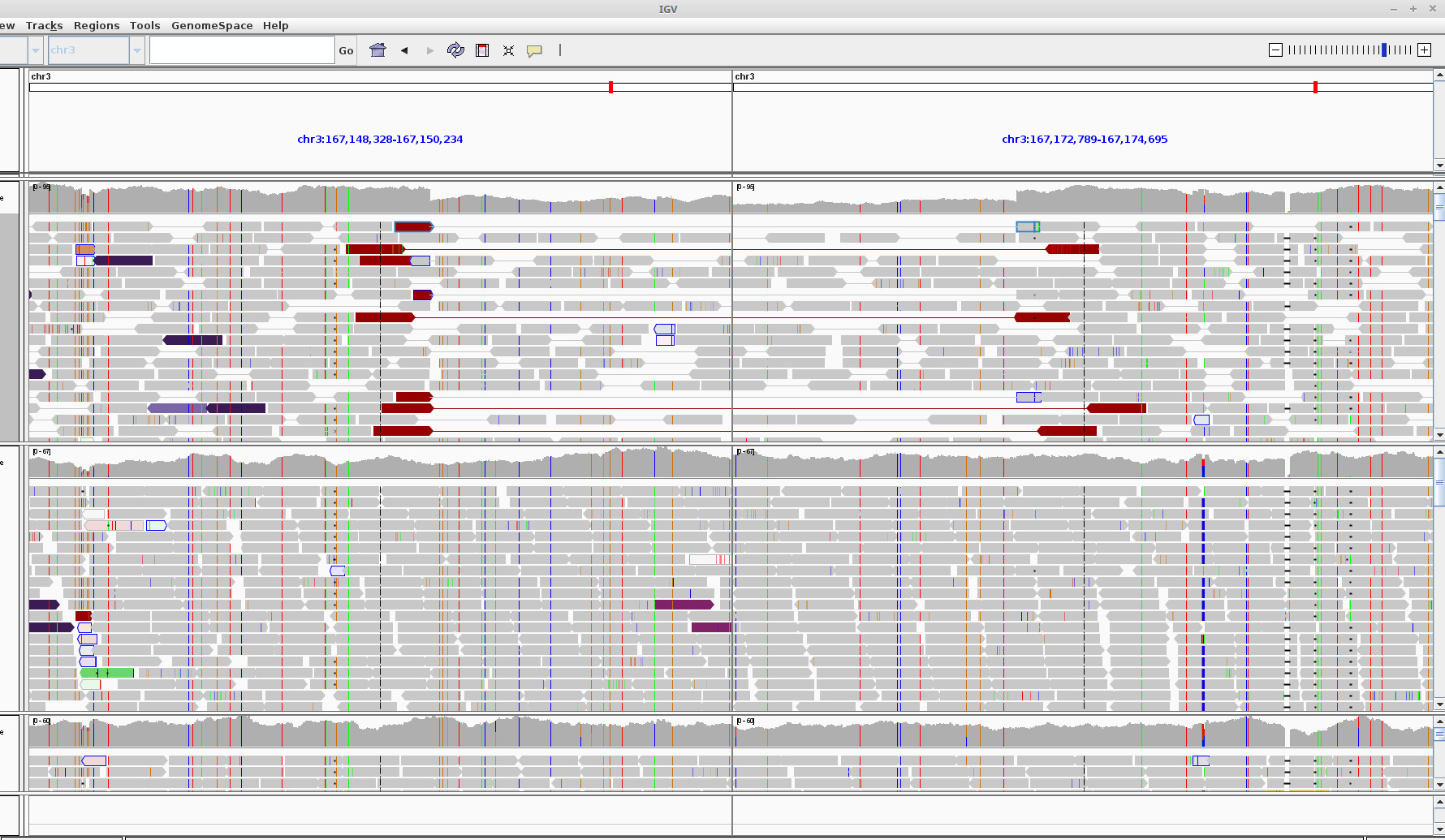

Supplement: msz176_Supplementary_Data [file msz176_supplementary_data.zip › msz176-Suppl_data/Supplementary_Figure S17.jpg]

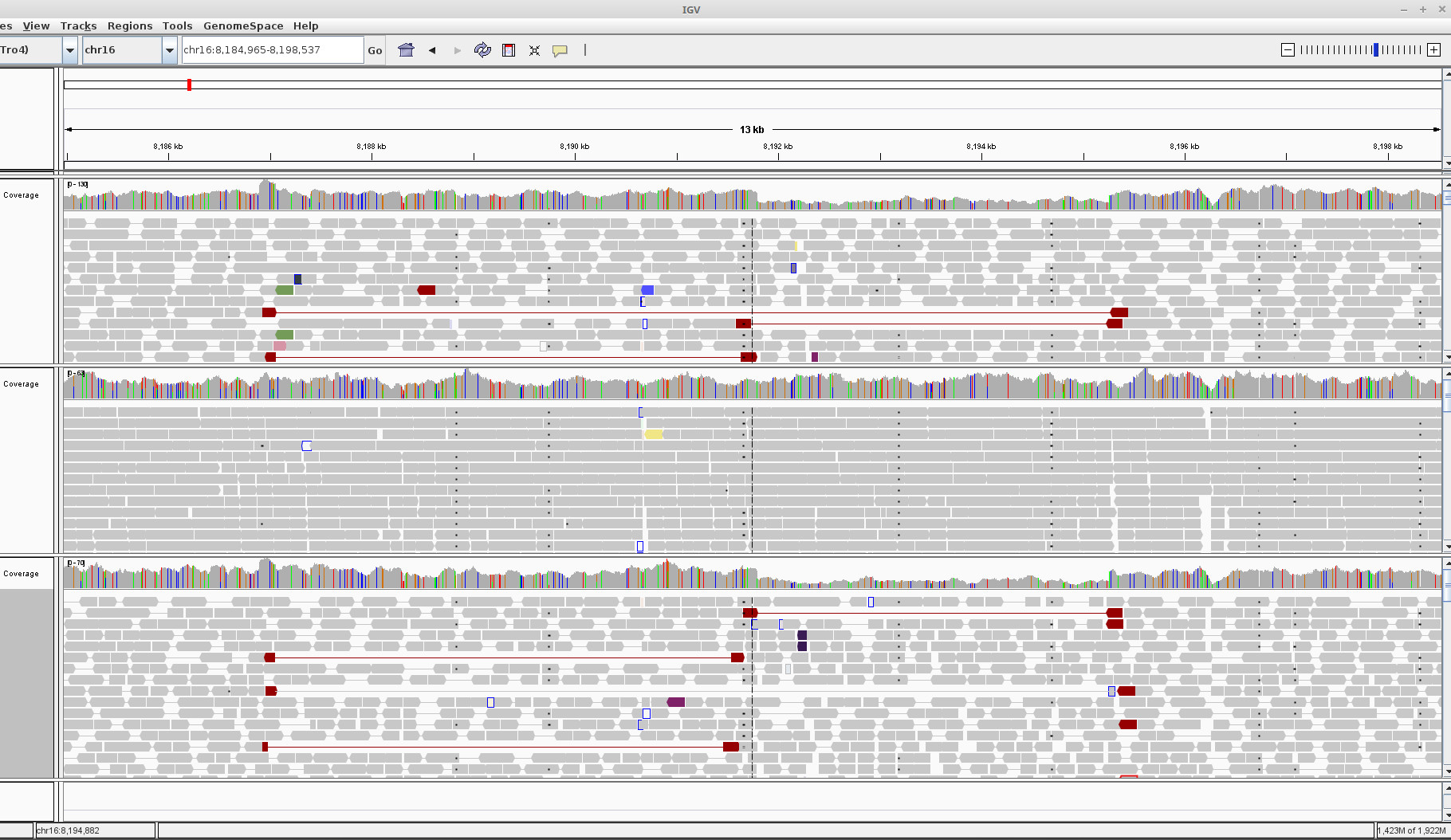

Supplement: msz176_Supplementary_Data [file msz176_supplementary_data.zip › msz176-Suppl_data/Supplementary_Figure S19.jpg]

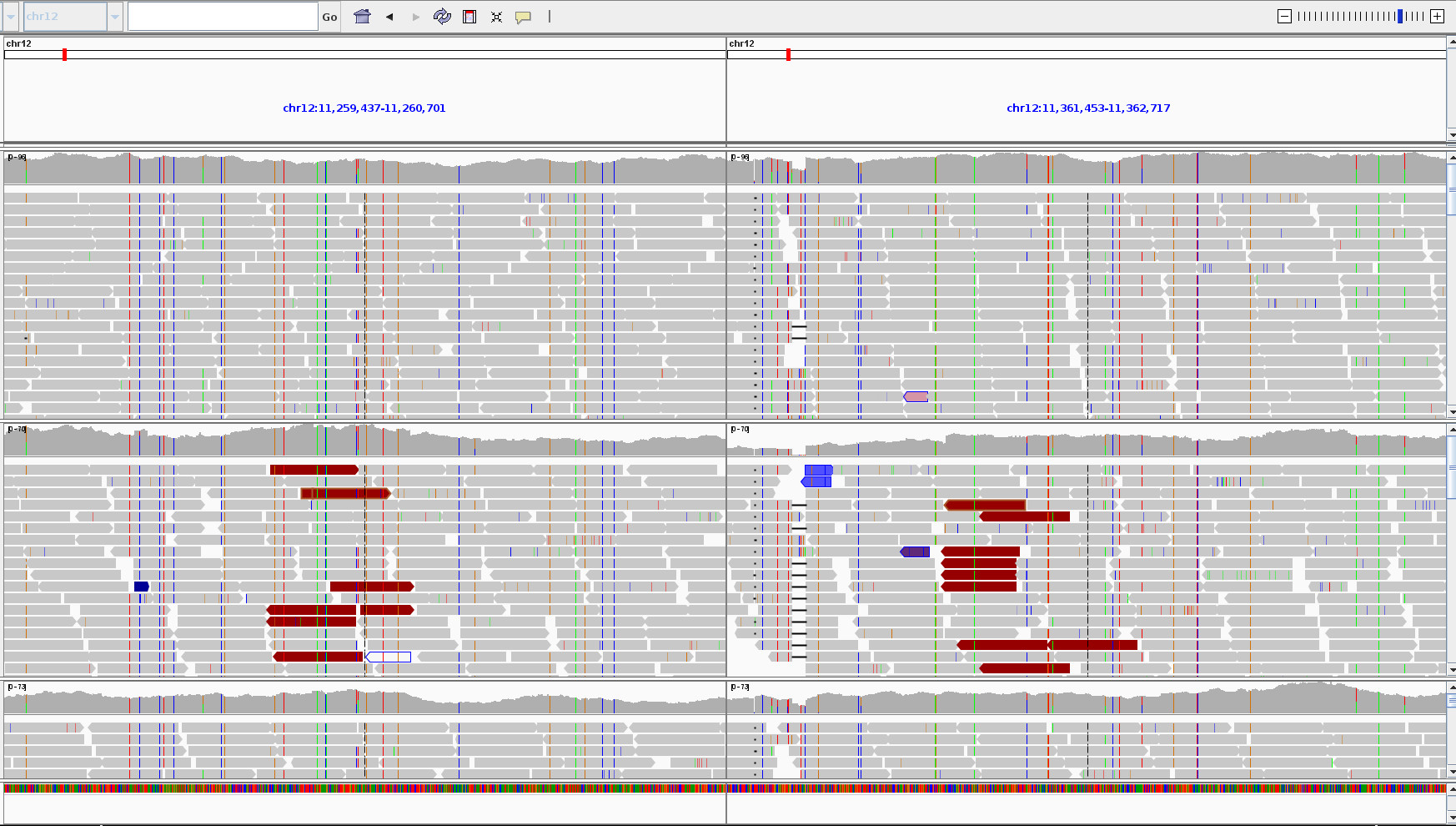

Supplement: msz176_Supplementary_Data [file msz176_supplementary_data.zip › msz176-Suppl_data/Supplementary_Figure S13.jpg]

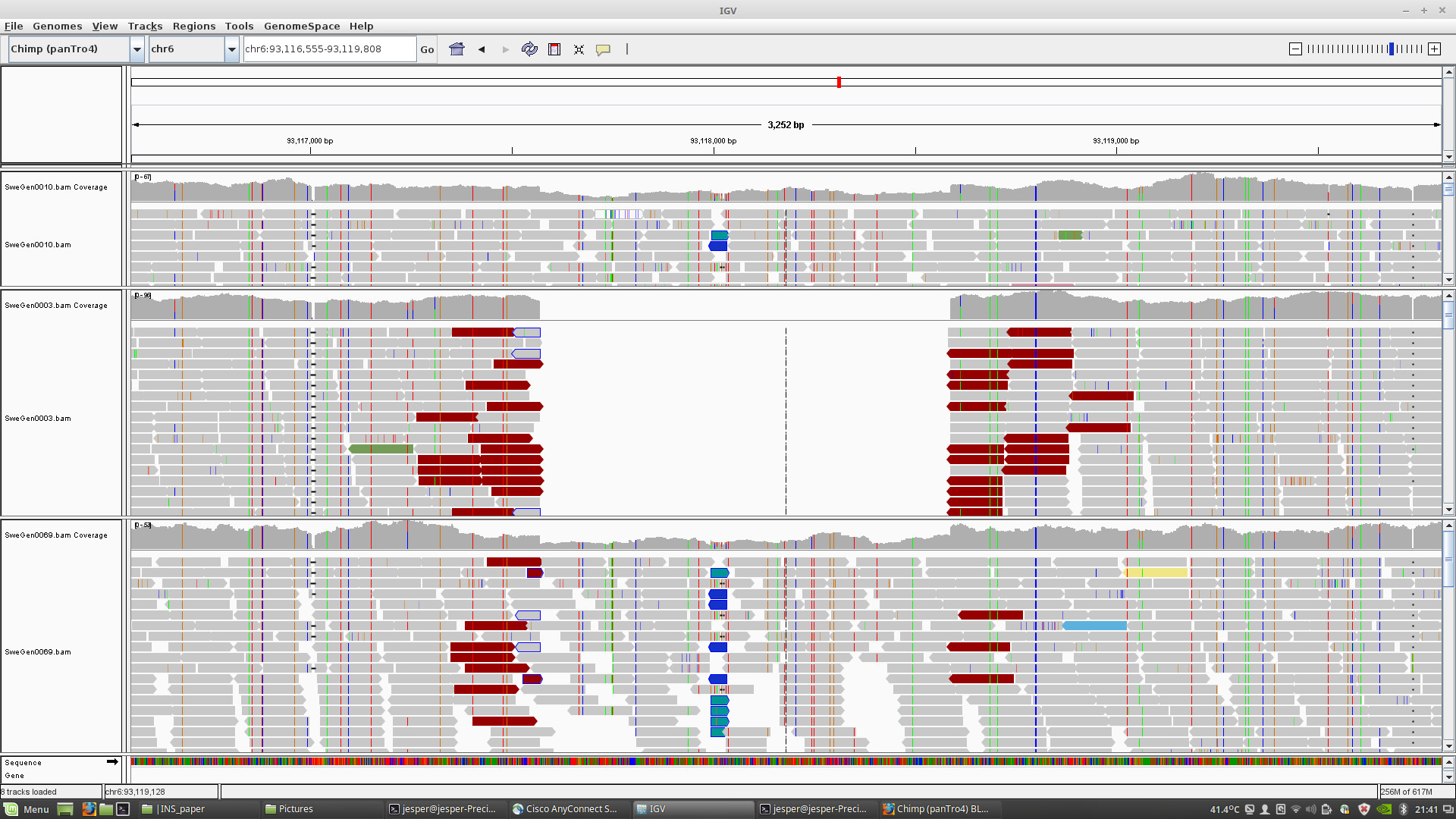

Supplement: msz176_Supplementary_Data [file msz176_supplementary_data.zip › msz176-Suppl_data/Supplementary_FIgure S5.jpg]

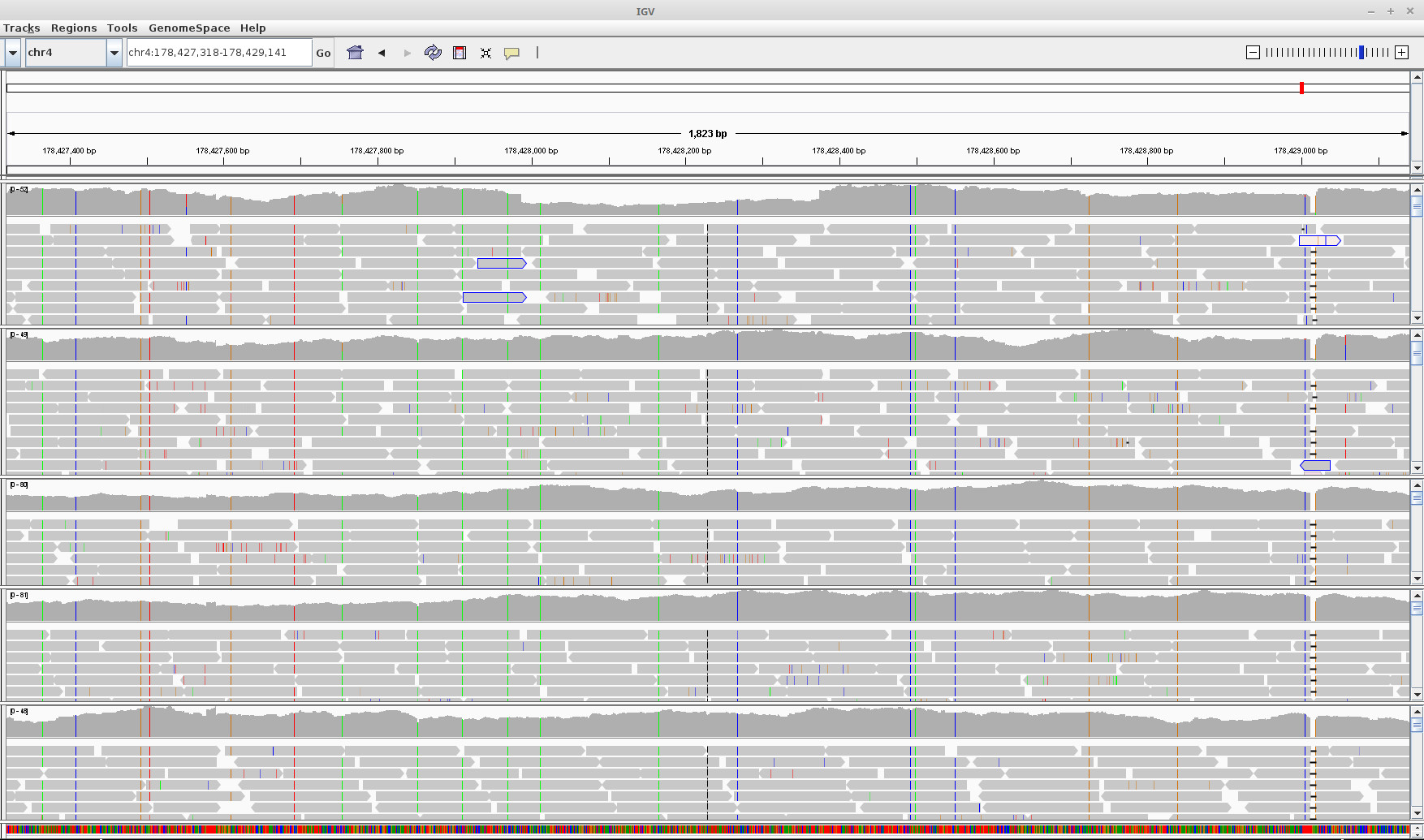

Supplement: msz176_Supplementary_Data [file msz176_supplementary_data.zip › msz176-Suppl_data/Supplementary_Figure S11.jpg]

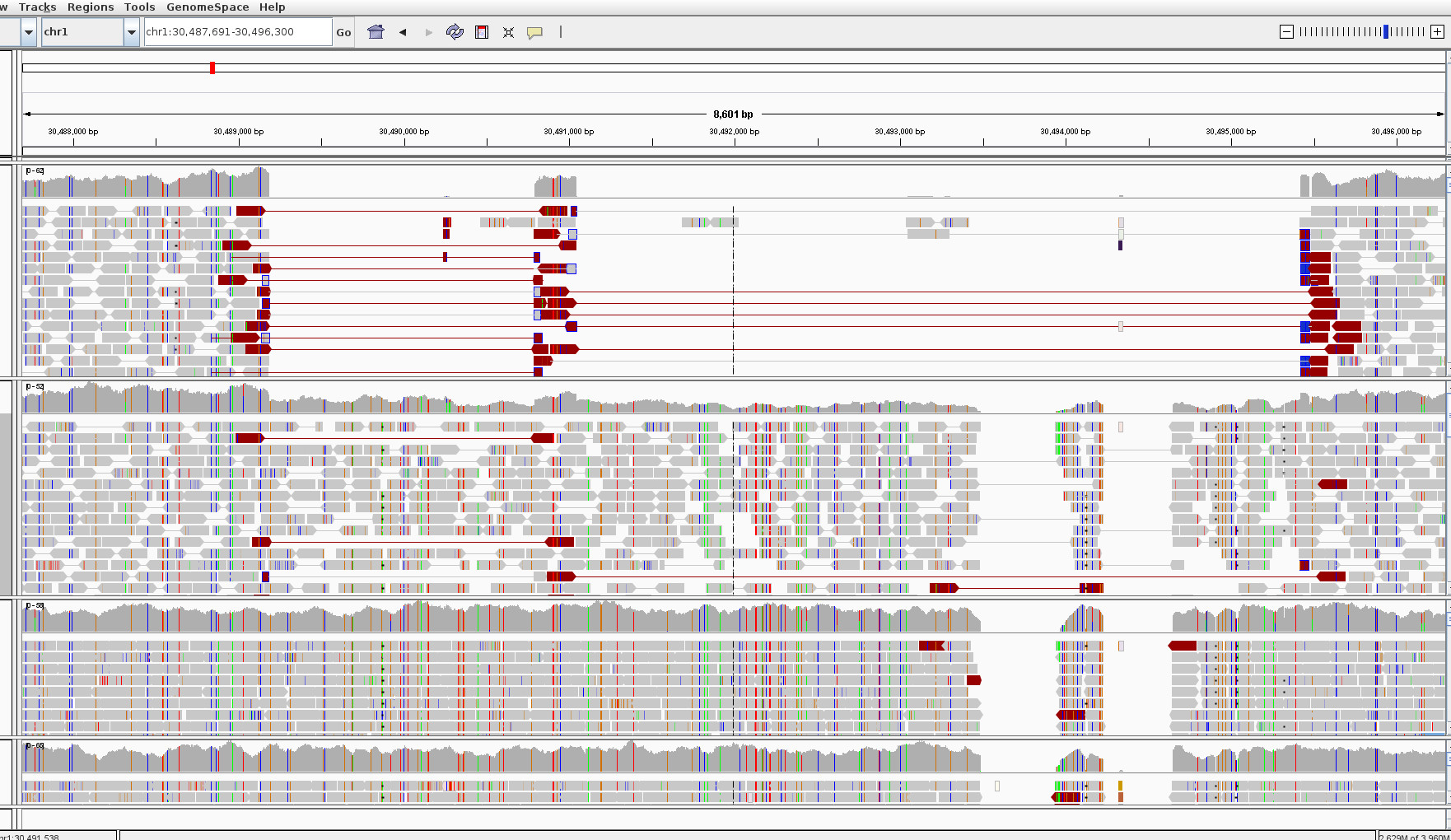

Supplement: msz176_Supplementary_Data [file msz176_supplementary_data.zip › msz176-Suppl_data/Supplementary_Figure S18.jpg]
